# SubCell: Proteome-aware vision foundation models for microscopy capture single-cell biology

**DOI:** 10.1101/2024.12.06.627299

**Published:** 2025-10-30

**Authors:** Ankit Gupta, Zoe Wefers, Konstantin Kahnert, Jan N. Hansen, Mohini K. Misra, Will Leineweber, Anthony Cesnik, Dan Lu, Ulrika Axelsson, Frederic Ballllosera, Russ B. Altman, Theofanis Karaletsos, Emma Lundberg

**Affiliations:** 1 Science for Life Laboratory, School of Engineering Sciences in Chemistry, Biotechnology and Health, KTH Royal Institute of Technology, Stockholm, Sweden.; 2 Bioengineering Department, Stanford University, Stanford, CA, USA.; 3 Computer Science Department, Stanford University, Stanford, CA, USA.; 4 Chan Zuckerberg Initiative, Redwood City, CA, USA.; 5 Pathology Department, Stanford University, Stanford, CA, USA.; 6 Chan Zuckerberg Biohub, San Francisco, CA, USA.

## Abstract

Cell morphology and subcellular protein organization provide important insights into cellular function and behavior. These features of cells can be studied using large-scale protein fluorescence microscopy, and machine learning has become a powerful tool to interpret the resulting images for biological insights. Here, we introduce SubCell, a suite of self-supervised deep learning models for fluorescence microscopy designed to accurately capture cellular morphology, protein localization, cellular organization, and biological function beyond what humans can readily perceive. These models were trained on the proteome-wide image collection from the Human Protein Atlas with a novel proteome-aware learning objective. SubCell outperforms state-of-the-art methods across a variety of tasks relevant to single-cell biology and generalizes to other fluorescence microscopy datasets without any fine-tuning. Additionally, we construct the first proteome-wide hierarchical map of proteome organization that is directly learned from image data. This vision-based multiscale cell map defines cellular subsystems with high resolution of protein complexes, reveals proteins with similar functions, and distinguishes dynamic and stable behaviors within cellular compartments. Finally, Subcell enables a rich multimodal protein representation when integrated with a protein sequence model, allowing for a more comprehensive capture of gene function than either vision-only or sequence-only models alone. In conclusion, SubCell creates deep, image-driven representations of cellular architecture that are applicable across diverse biological contexts and datasets.

## 1 Introduction

As a fundamental unit of life, cells have a remarkable capacity to perform a wide variety of functions by adopting diverse morphologies and carefully orchestrating a multitude of biological processes simultaneously. Proteins and other biomolecules localize to specific cellular substructures, undergo shifts in localization, and are subject to local modifications that enable the cell to adapt its function and identity. Protein localization and expression are highly variable, and often disease-causing mutations lead to mislocalizations that coincide with changes in cell morphology [1–4]. Therefore, understanding cell behavior requires comprehensive spatial mapping of cell morphology, subcellular architecture, and dynamics, which can currently only be captured at scale by microscopy. Consequently, fluorescence microscopy has become an indispensable tool for systematically characterizing protein localization [5–7] and for profiling morphological responses to chemical and genetic perturbations [8–14] at scale.

The massive scale of high-throughput fluorescence microscopy datasets requires more automated analysis methods. Profiling tools [3] and machine learning approaches [15–19] that create single-cell feature embeddings have demonstrated faster and deeper analysis of large image datasets beyond human perception. Self-supervised learning has emerged as a particularly powerful paradigm for general feature representation in biology [20–22]; however, bespoke models are typically trained separately for each dataset and for different biological tasks. The proliferation of machine learning models with narrow applicability to different datasets has created the critical need for an easy-to-use feature extractor model capable of matching the performance of task-specific models across different imaging modalities.

Here, we present SubCell, a suite of foundational vision models for representing single cells from fluorescence microscopy. Trained on the proteome-wide pattern diversity of the Human Protein Atlas (HPA) [5], SubCell utilizes a multitask learning framework to efficiently encode both cellular phenotypes and subcellular organization. Without fine-tuning, SubCell produces robust representations of cell morphology and protein localization across diverse independent datasets that vary greatly in image resolution, channel markers, cell types, and even species. We demonstrate that SubCell supports a wide range of downstream tasks, including localization classification, cell cycle modeling, drug response prediction, and mechanism-of-action identification. Finally, by capturing subtle, continuous patterns of protein localization, SubCell enables the creation of the first image-based multiscale map of subcellular protein organization and enriches protein sequence representations for functional genomics, facilitating a systems-level understanding of proteins and cells.

## 2 Results

### Building proteome-aware models for single-cell images

**Fig. 1:**
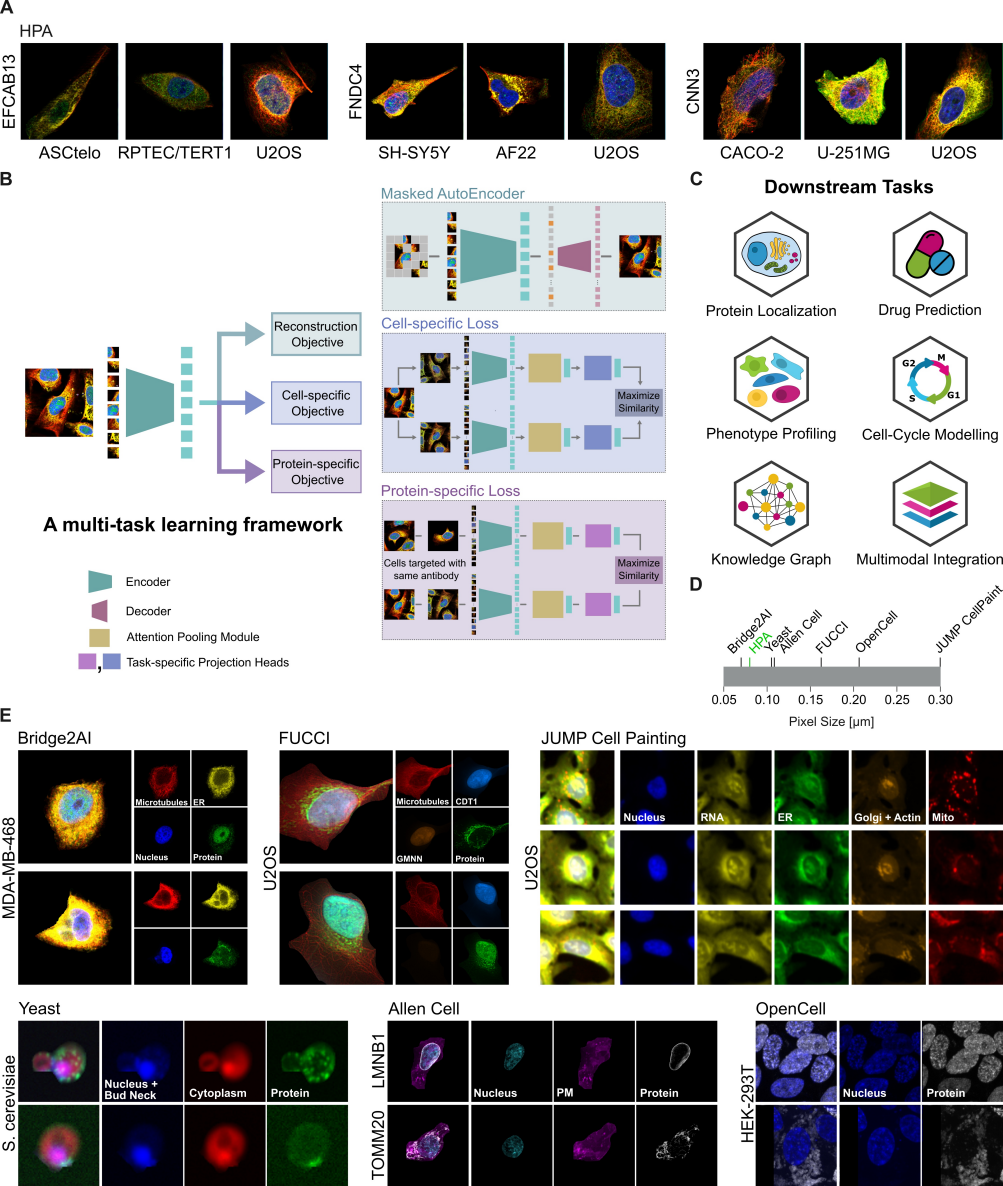
Overview of the SubCell self-supervised learning framework. (A) Representative images of single-cell crops from the Human Protein Atlas dataset demonstrating the diverse cell morphologies and protein subcellular localizations present in a wide range of human cells (blue: nucleus; red: microtubules; yellow: endoplasmic reticulum; green: protein of interest). The images show three different proteins (EFCAB13, FNDC4 and CNN3) in a variety of human primary and cancer cell lines derived from different organs of origin in donors of different age and sex (ASC52telo: mesenchyme, adipose tissue; RPTEC/TERT1: epithelium, kidney; U-2 OS: osteosarcoma; SH-SY5Y: brain; AF22: neuroepithelial stem cells; CACO-2: adenocarcinoma; U-251MG: glioblastoma (more details see Materials and Methods)) (B) Illustration depicting our multi-task learning approach to train vision transformer models. We use three objectives to train our models: reconstruction, cell-specific, and protein-specific objectives. (C) Examples of the downstream tasks that are enabled with SubCell shown in this work. (D) The pixel size of each of the datasets used for evaluating SubCell (HPA, highlighted in green, was used for training). (E) Representative images from the datasets used in the evaluations to showcase diversity in resolution and imaging channel: (top, left to right) Bridge2AI, FUCCI U2OS, and JUMP cell paint datasets, and (bottom, left to right) yeast cell cycle, Allen Cell, and OpenCell datasets.

SubCell was trained as a generalizable vision model using fluorescence microscopy images that capture a broad range of cellular characteristics, including protein localization patterns and cell morphology. Since proteins are key structural and functional molecules in cells, we reasoned that a proteome-based training framework would enable the model to learn attention patterns relevant to cellular function. For this purpose, we used the HPA (v23) dataset, which contains images of 1.13 million single cells, encoding the expression and subcellular distribution of 13,141 proteins across 37 human cell lines representing cells of different developmental origins and tissue of origin with vastly different phenotypes (e.g., suspension cells, epithelial cells, fibroblasts). The HPA was chosen due to the great diversity of cell lines, protein subcellular localization patterns, and high image resolution (Fig. 1A).

We examined various state-of-the-art unsupervised learning methods, including masked autoencoders (MAEs), and self-supervised learning techniques such as contrastive learning and self-distillation. We found that these techniques learned morphology well but fell short in encoding protein subcellular localization patterns (See Methods and Supplementary). In addition to the standard reconstruction objective, we introduced the concepts of “cell-specific” and “protein-specific” objectives (Fig. 1B), which guide the models with biologically relevant information about the images (see Methods). The cell-specific objective enabled the model to learn features of cell line identity, whereas the protein-specific objective helped the model understand protein localization. We combined the three objectives in a multitask learning framework (Fig. 1B, detailed in Supplementary Fig. S1) to train a vision transformer (ViT) model [23] with 86.4 million parameters, thus achieving a generalized representation of single-cell images. We added an attention-pooling module to the encoder’s output to prevent the model from learning spurious background features and optimized the multitask learning framework by evaluating combinations of learning objectives. The best-performing SubCell models were ViT-ProtS-Pool, trained solely with the protein-specific objective, and MAE-CellS-ProtS-Pool, trained with the multitask objective of combining reconstruction, cell-specific, and protein-specific losses. These two models were further explored for various downstream tasks and in various microscopy datasets (Fig. 1C-E).

### SubCell robustly profiles protein localization and cell cycle dynamics

#### Accurate mapping of protein localization across diverse cell types

**Fig. 2:**
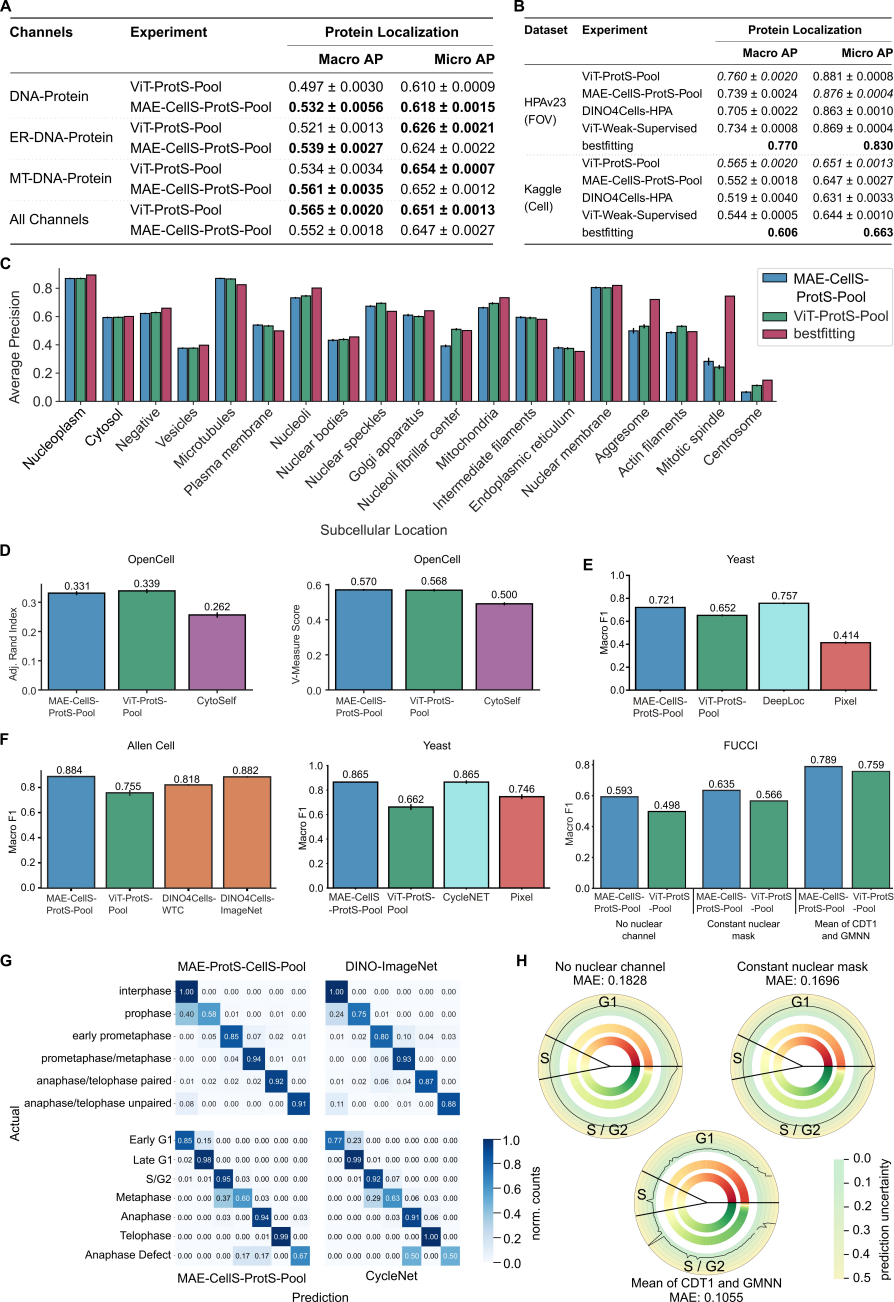
Quantitative evaluation of learned representations for predicting protein localization and cell cycle stages across different datasets. (A) Evaluation of our models trained with different channel combinations for protein localization prediction and cell-line classification on the Kaggle test set. (B) Results of protein localization prediction on the HPA test set (FOV level) and hidden Kaggle test set (single-cell level), showing overall macro and micro average precision. In both tables, the best performance for each metric is highlighted in bold; for the table in A, the second-best is additionally highlighted in italics. (C) Protein localization results by category on the Kaggle test set. Bar plots show the average precision for each category for bestfitting (magenta), ViT-ProtS-Pool (green), and MAE-CellS-ProtS-Pool (blue). (D) Clustering performance of the models on the OpenCell dataset, evaluated using adjusted Rand index (left) and V-measure score (right). (E) Classification performance on the yeast cell dataset, with bar plots showing the macro F1-score. (F) Macro F1-scores for cell cycle stage classification across three datasets: Allen Cell dataset (left, six stages), yeast dataset (middle, seven stages), and FUCCI U2OS dataset (right, three stages). For the FUCCI dataset, results are shown for different nuclear stain replacements: no nuclear channel, a binary nuclear mask with constant intensity, and the mean of the CDT1 and GMNN channels. (G) Confusion matrices of the two best classification models in the AllenCell dataset (top) and the yeast dataset (bottom), showing the actual vs predicted normalized counts for the cell cycle stages. (H) The plots display the results of the pseudotime regression model for the three different configurations. The innermost circle represents the true pseudotime, while the middle circle depicts the predicted pseudotime, where the colors indicate the pseudotime value, ranging from zero (red) to one (green). The outermost circle represents the uncertainty bands, and the black line indicates the uncertainty in the prediction of the trained models at a particular pseudotime. The missing sections in the line indicate the regions where the uncertainty is beyond the plotting range.

SubCell was trained on HPA images that consist of three cellular reference markers — nucleus (DNA), endoplasmic reticulum (ER), and microtubules (MT) — as well as a protein of interest (Fig. 1A). We trained additional versions of the ViT-ProtS-Pool and MAE-CellS-ProtS-Pool models with different combinations of the three reference markers to test their relative importance for protein localization (Fig. 2A). Interestingly, protein localization performance slightly improved when the ER marker was removed, worsened upon removal of the MT marker, and was the worst when only DNA was used as a reference marker (Fig. 2A). These results highlight the versatility of SubCell in working with different channel combinations, but also demonstrate the need to systematically evaluate reference markers beyond those used in the HPA to help determine which set best supports model development for protein localization classification.

We next benchmarked SubCell’s protein localization performance against current state-of-the-art models, which were also trained on images from the HPA dataset [16, 24], as well as a weakly supervised baseline model with the same architecture as SubCell. We evaluated the performance on an HPA test set and the hidden Kaggle challenge test set [24] (Fig. 2B). Model performance was assessed at the field-of-view (FOV) level for HPA data and at the single-cell level for the hidden Kaggle dataset. In both datasets, SubCell outperformed the self-supervised DINO4Cells-HPA model [16] and the weakly supervised ViT model with the same architecture as SubCell. The supervised “bestfitting” model [24], which was the winner of a Kaggle challenge focused on predicting protein localization patterns in the HPA dataset, narrowly outperformed the ViT-ProtS-Pool model in the Kaggle dataset, but SubCell had a higher micro AP in the HPA set. A breakdown of protein localization categories showed that “bestfitting” outperformed SubCell mainly in rare classes, such as the mitotic spindle and centrosome, for which the “bestfitting” model employed additional techniques to boost performance (Fig. 2C).

SubCell’s performance was next tested on two additional datasets containing different channels and pixel sizes from those on which SubCell had been trained: the OpenCell dataset [6] and a yeast dataset [25]. For the OpenCell dataset, the DNA-Protein version of SubCell outperformed a self-supervised model trained on the dataset, CytoSelf [19] (Fig. 2D). For the yeast dataset, we compared the MT-DNA-Protein version of SubCell against DeepLoc [25], a supervised model trained on the dataset for protein localization classification, and a multilayer perceptron (MLP) classifier trained directly on the image pixels. SubCell-based classifiers significantly outperformed the pixel MLP, but not DeepLoc (Fig. 2E). This showed that SubCell extracted meaningful semantic features beyond pixel values, even on images with little resemblance to the training data.

#### Precise characterization of cell cycle states

To showcase SubCell’s capabilities beyond protein localization, we evaluated its ability to capture morphological changes related to cell cycle dynamics from datasets not included during training: human induced Pluripotent Stem Cell (hiPSC) (AllenCell dataset) [7], yeast [25], and FUCCI-U2OS [1] (see Methods for dataset details). For the AllenCell dataset, we tried different marker combination versions of the models (Supplementary Table S1) and report the best here. The MAE-CellS-ProtS-Pool model outperformed two DINO models, one trained on the AllenCell dataset and the other trained on ImageNet [26] (Fig. 2F, left barplot) [16]. For the yeast dataset, the MT-DNA-Protein version of MAE-CellS-ProtS-Pool matched the performance of the supervised CycleNet model and outperformed the pixel-based model trained to classify cell cycle stages (Fig. 2F, middle barplot) [25]. Notably, the best results for the MAE-CellS-ProtS-Pool model were obtained using only a logistic regression classifier (Supplementary Table S2), suggesting that the embedding space learned by the model linearly separates cell cycle classes and is rich in morphological information (see Supplementary Fig. S2). The confusion matrices (Fig. 2G) revealed that SubCell struggled primarily to distinguish between interphase and prophase in the AllenCell dataset and between S/G2 and metaphase in the yeast dataset.

Finally, the FUCCI dataset was used to assess SubCell’s capability to predict the cell cycle in the absence of the nuclear (DNA) marker. To that end, various options were tested to replace the channel, including a blank channel, a channel with a constant value for the nuclear mask derived from the FUCCI markers CDT1 and GMNN, which are also nuclear, and a nuclear channel constructed as the mean of the FUCCI markers. The FUCCI classification tasks demonstrated that SubCell effectively predicted cell cycle stages using the latter approach (Fig. 2F, right barplot). SubCell also predicted the whole cell cycle pseudotime with an average absolute error of 0.105, although the uncertainty in the prediction was high at the beginning and end of the cell cycle (Fig. 2H). The early G1 and G2/M entry stages represent morphologically “in-between” states, where nuclear and cytoskeletal rearrangements are occurring, reducing the separability in feature space and leading to increased uncertainty in those phases [1]. The performance of ViT-ProtS-Pool was significantly lower than that of the multitask model for the cell cycle prediction tasks. This can be attributed to the fact that it was trained only with the protein-specific objective, making it inherently less focused on morphology, which informs the cell cycle stages within the cell.

The results demonstrate that SubCell generalizes to unseen datasets of different resolutions and even vastly different appearances (yeast). SubCell accurately classifies protein localization and cell morphology patterns without fine-tuning, performing better or on par with models trained explicitly on the datasets.

### SubCell encodes biologically relevant features

#### Unbiased profiling of cellular morphology and localization hierarchy

**Fig. 3:**
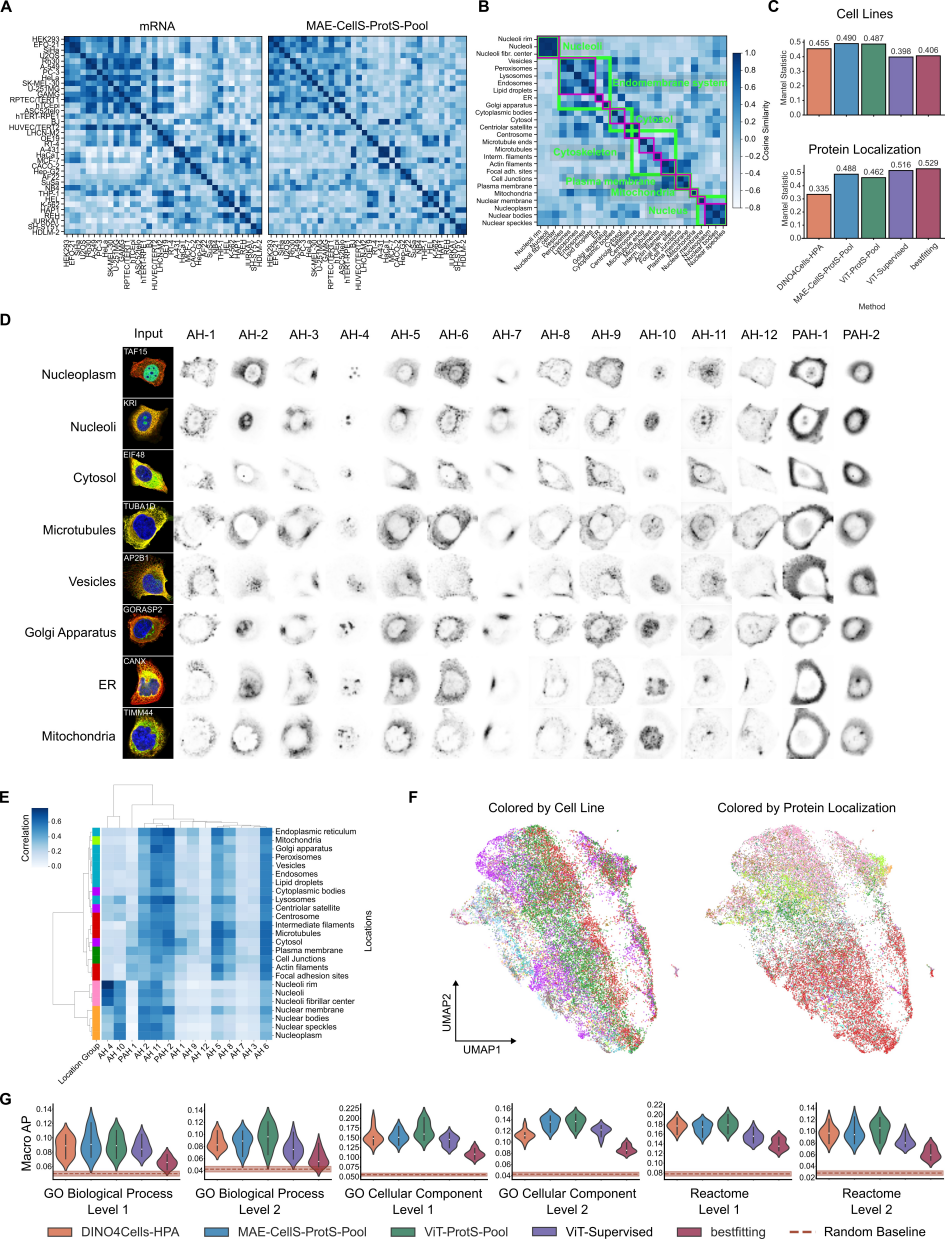
Feature validation of SubCell. (A) Profiling of the cell lines using bulk mRNA levels (left) and aggregated MAE-CellS-ProtS-Pool features (right). The matrices show the cosine similarity between cell lines based on mRNA levels or model features. The order of rows and columns follows the groups determined by the hierarchical clustering of the mRNA similarities. (B) Cosine similarity among the MAE-CellS-ProtS-Pool features aggregated by localization categories. Clusters highlighted in green are the localizations in seven major categories, and the clusters highlighted in magenta are the minor categories. (C) Mantel statistic for the correlation between cell line aggregated feature profiles from SubCell embeddings and the associated bulk mRNA sequencing profiles (top) and subcellular localization patterns from SubCell and the hierarchical profiles annotated by experts (bottom). (D) Examples of fluorescent images and the attention captured by the attention heads and pooled attention heads in the MAE-CellS-ProtS-Pool model for different protein localizations. The twelve attention heads of the vision transformers are marked by the prefix AH-, and the pooled attention heads by the prefix PAH-. (E) A cluster map showing the aggregated correlation profiles of the attention heads with the protein channel for different localization categories. The order of rows and columns is set by hierarchical clustering of the correlation profiles. The positions of major location groups are shown in different colors on the left side of the plot. (F) UMAP visualization of the correlation profiles of all the fluorescent channels with the attention maps for the HPA dataset. The profiles were aggregated over the FOVs, and the colors represent cell lines (left) and protein localization groups (right). (G) Violin plots showing the macro-AP score comparison of the classification performance of the models across different known databases. Level 1 and Level 2 designations relate to the hierarchical granularity of the dataset annotations, with Level 1 related to higher-level groupings while Level 2 is more granular (see Methods).

SubCell’s encoding of meaningful morphological information was further evaluated using an approach similar to that of Doron et al.[16]. Morphological profiles of the 36 cell lines were created from the single-cell embeddings and clustered by cosine similarity, which closely resembled similarities determined from bulk mRNA sequencing (Fig. 3A). Aggregation of SubCell embeddings by protein localization closely matched hierarchical groupings of 26 organelles (see details in Supplementary Table S3), which were also produced by expert annotations (Fig. 3B). SubCell outperformed DINO4Cells-HPA and supervised models in similarity to the mRNA sequencing results (Fig. 3C, top), and outperformed DINO4Cells-HPA in protein localization similarity (Fig. 3C, bottom). These results demonstrate that SubCell encodes features of cell line characteristics (i.e., morphology) and subcellular architecture better than existing self-supervised models and comparably to supervised models.

#### Biologically meaningful subcellular attention

We assessed the underlying transformer’s attention pooling module to examine which parts of an image SubCell learned that capture biologically meaningful information. ViTs convert an image into a sequence of patches and leverage an attention mechanism between them to learn complex features. The attention generated for each patch provides valuable insights into the model’s behavior and decision-making process. Fig. 3D shows the attention captured by the twelve attention heads and two pooled attention heads for various protein localization patterns for the MAE-CellS-ProtS-Pool model. Interestingly, most attention heads were directed to specific subcellular regions, many resembling patterns of organelles; for example, AH-4 to nucleoli, AH-8 to microtubules, PAH-2 to the endoplasmic reticulum, AH-12 to the plasma membrane, and AH-10 to the nucleoplasm.

The relationship between the attention heads and the input cell was assessed by computing the correlation between the different channels of an image and the attention weights from each of the attention heads. This resulted in a 14-dimensional vector for each channel, which we refer to as an attention-correlation profile. We aggregated these attention-correlation profiles for the protein channel (see Methods) over different localization categories and performed hierarchical clustering to see how the profiles are organized (Fig. 3E). The resulting cluster map recapitulated the major localization clusters defined in the previous section (Fig. 3B), indicating that the learned attention patterns reflect biologically meaningful subcellular compartmentalization. UMAP visualization of the attention-correlation profiles across all channels for the whole HPA dataset also revealed interesting patterns in the attention learned by the model (Fig. 3F). The major axes of variation in the profiles aligned orthogonally for the protein localizations (vertically) and cell phenotype (horizontally). Altogether, these results demonstrate that the model learned biologically meaningful relationships and that its attention mechanisms effectively disentangled morphological information (cell line) from subcellular patterns. A similar plot for the ViT-ProtS-Pool model is shown in Supplementary Fig. S3.

#### Protein relations across known biology

Having established that SubCell learns features relevant to protein localization and cell morphology, we next asked how well it captures known biological relations. Drawing from hierarchical levels (see Methods) of the Reactome [27], Gene Ontology (GO) cellular compartment (CC), and biological process (BP) databases [28], we found that SubCell outperformed both supervised and unsupervised models in predicting biologically relevant protein groupings (Fig. 3G). This challenging task, which is impossible to perform with the human eye, indicates that SubCell encodes known biological relationships more effectively than existing vision models. The overall low performance across all models can be attributed to the difficulty of the task, as well as several sources of noise, including sparse and cell-type-agnostic databases, and the limited information that images cannot reasonably provide about interactions and sequences.

### SubCell predicts changes in localization and morphology in response to drug perturbation

#### Predicting drug perturbations in breast cancer cells

**Fig. 4:**
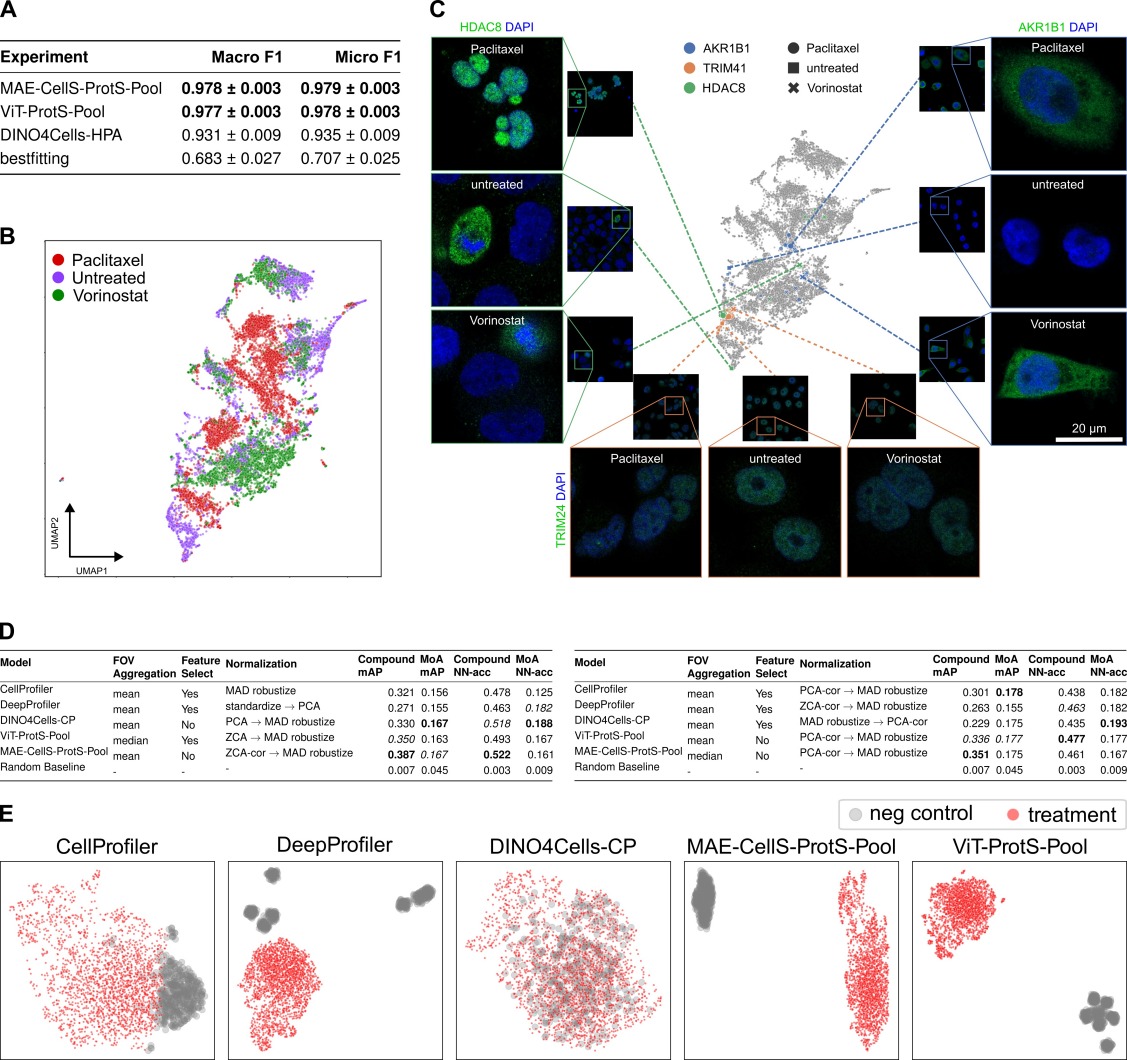
Evaluation of morphology predictions in the drug perturbed datasets. (A) The results of 10-fold cross-validation for drug prediction on the single-cell features extracted from the different models. The best performance for each metric is highlighted in bold, and the second best is highlighted in italics. (B) A vignette showing the UMAP plot of the FOV features and the FOV images highlighting different effects of the drugs captured by the MAE-CellS-ProtS-Pool model on three genes, namely, HDAC8, TRIM24, and AKR1B1. (C) UMAP plot of the FOV features highlighting the different treatments with Paclitaxel (red), untreated (violet), and Vorinostat (green). (D) The results of the post-processing pipeline for each model that resulted in the best compound mean average precision (mAP) (left) and the best MoA mAP (right). We also report the nearest-neighbor accuracy (NN-acc) for both replicate retrieval and MoA prediction. For both tables, the best performance for each metric is highlighted in bold, and the second best in italics. (E) UMAP visualizations of the post-processed well-level profiles for each model that resulted in the best MoA mAP, colored by treated (red) vs control(grey).

To further explore the generalization capabilities of SubCell in novel use cases and cell types, we investigated its performance in quantifying and predicting drug effects. This task was assessed using the CM4AI dataset of triple-negative breast cancer cells treated with the anti-cancer drugs Vorinostat or Paclitaxel [29]. Images of 454 different proteins were acquired in the HPA format. SubCell classified drug treatments considerably better than the bestfitting and DINO models (Fig. 4A).

SubCell’s capacity to detect drug-induced changes was further assessed by inspecting the feature space. UMAP visualization of how cell embeddings were distributed in the feature space revealed clustering by treatment (Fig. 4B). Proteins that maintained a stable localization upon drug treatment, like TRIM24, exhibited short distances in the learned feature space between untreated and drug-treated conditions (Fig. 4C) (average cosine distances: 0.027 (untreated vs. Paclitaxel) and 0.041 (untreated vs. Vorinostat)). In contrast, the translocation of HDAC8 from the cytosol to the nucleoplasm upon Paclitaxel treatment coincided with a cosine distance of 0.158. Vorinostat treatment resulted in a cosine distance of 0.119 from the untreated cells for HDAC8, indicating that changes in protein localization and morphology affected the distance in the embedding space more than just a morphological change. The relatively high overall distance compared to the TRIM24 images may reflect that the single-cell variation in protein localization for HDAC8 spreads the data points for individual fields of view more in feature space than if one looks at images with stable localization across cells, as for TRIM24 (Fig. 4C, small symbols). The SubCell embeddings also reflected changes in expression level differences between untreated and treated conditions, such as AKR1B1 (cosine distances: 0.130 (untreated vs. Paclitaxel) and 0.116 (untreated vs. Vorinostat)). Despite AKR1B1 localizing to the cytoplasm in both Paclitaxel and Vorinostat treatments, overall cellular morphology differed; we observed a rounder morphology and less single-cell variation in protein expression level with Paclitaxel compared to Vorinostat treatment, leading to a still remarkable distance in embedding space. We conclude that SubCell readily generalizes to new cell types not present in the training data, including drug-treated cells with perturbed morphology. The accurate classification of drug treatments reflects SubCell’s ability to detect changes in protein localization, morphology, and expression level.

#### Morphological profiling from drug perturbation (Cell Paint) screens

SubCell’s ability to capture phenotypic effects of chemical and genetic perturbations was further tested with Cell Painting images from the JUMP1 dataset [9]. We compared our models to the three state-of-the-art single-cell methods used for Cell Painting data: CellProfiler [3], a bespoke pipeline consisting of handcrafted features; DeepProfiler [30], a weakly-supervised model trained to predict compound treatment labels; and DINO4Cells-CP [16], a self-supervised model trained on the JUMP dataset. We used the ER-DNA-Protein version of SubCell because it includes two common channels (ER, DNA) in the Cell Painting data (see Methods). We tested how SubCell performed on the JUMP1 dataset with two standard tasks: replicate retrieval, which involves identifying wells treated with the same compound across experimental batches, and mechanism of action (MoA) identification, which involves identifying compounds that affect the same biological mechanism.

SubCell excelled in replicate retrieval, with MAE-CellS-ProtS-Pool achieving the highest compound mean average precision (mAP) of 0.387, significantly better than the other models trained explicitly on the Cell Painting data (Fig. 4D). In contrast, the performance gap for the mechanism of action (MoA) prediction task was narrower (Fig. 4D). UMAP visualization of SubCell embeddings revealed better separation between the treated and control wells compared to CellProfiler (Fig. 4E). The strong performance of SubCell is particularly notable because, unlike DINO4Cells-CP and DeepProfiler, it was never trained on Cell Painting data and therefore was naive to the unseen imaging channels. We conclude that SubCell effectively captures the morphological features of perturbed phenotypes from different channels and at various resolutions. We expect fine-tuning and training of our models using lower-resolution images to further enhance their performance.

### SubCell enables creation of vision-based proteome-wide multiscale cell maps

Spatial proteomics based on high-resolution confocal imaging, as performed in the subcellular section of HPA, reveals the subcellular location of a protein at the resolution limit of light diffraction. In the HPA, 35 high-level subcellular organelles or compartments have been annotated. However, it is essential to acknowledge the existence of additional known and possibly unknown subcellular compartments, condensates and protein complexes. Recently, we demonstrated a computational framework for building a multiscale integrated cell map (MuSIC) that combined image and protein-protein interaction data to identify protein assemblies and novel subcellular complexes [31, 32]. However, this MUSIC model was limited to fewer than 5,000 proteins due to the sparsity of protein-protein interaction data. Given that SubCell has learned subtle and biologically relevant features, we hypothesized that it could be used to construct a vision-only multiscale map of the human proteome.

#### Defining cellular substructures with protein complex resolution

**Fig. 5:**
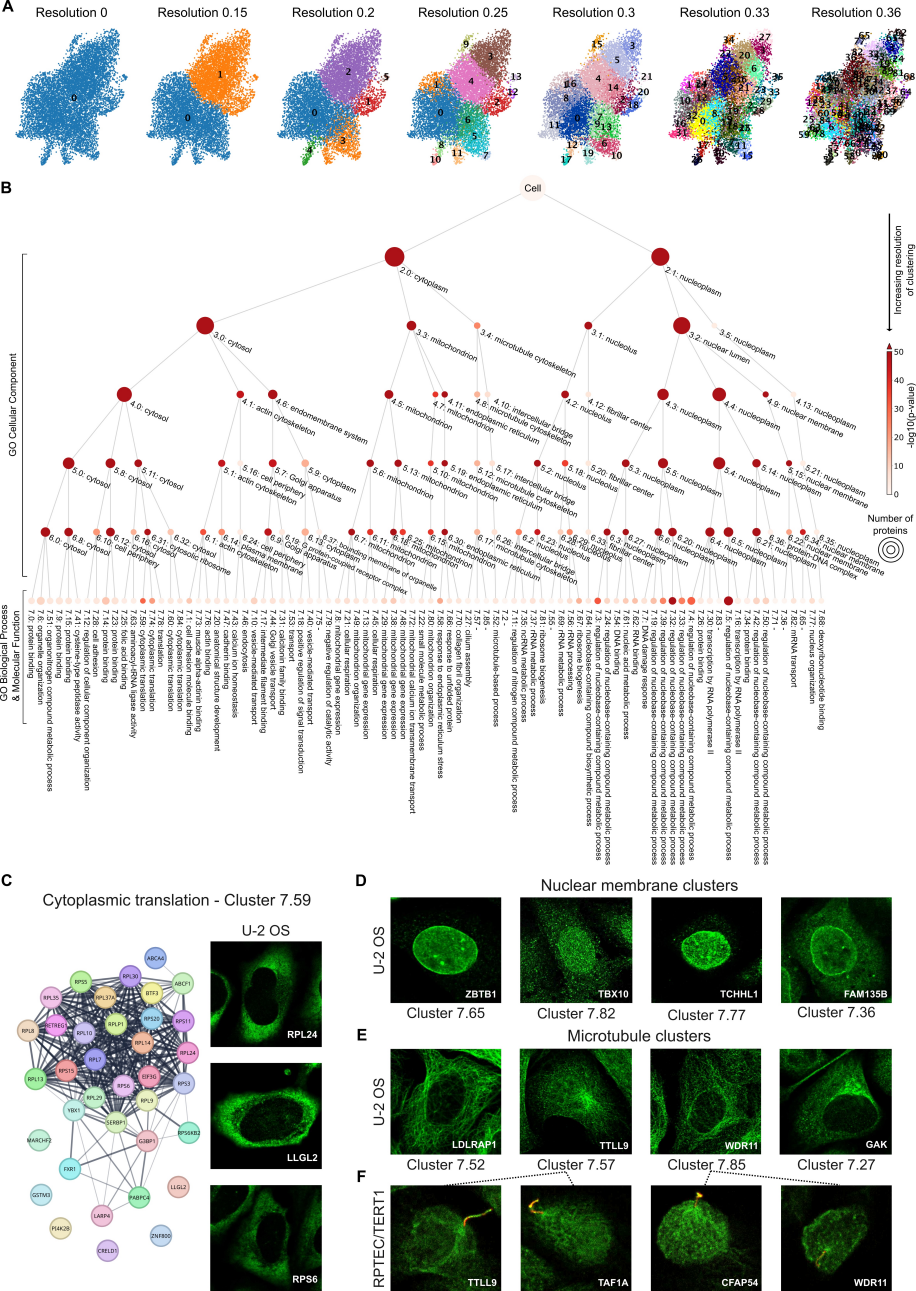
A vision-based multiscale map of cellular protein architecture in U-2 OS cells. (A) HPA protein embedding UMAPs showing the resulting clusters of Leiden subclustering at increasing resolutions. (B) Multiscale hierarchical map obtained by subclustering of the 9543 protein representations of HPA images. Clusters and subclusters were annotated by GO Cellular Compartment (levels 1–6) or GO Biological Process / Molecular Function (level 7) enrichment analysis; the most significant term is shown. Each node is a cluster from A at the given subclustering / resolution level. The node size indicates the number of proteins in the cluster; the node color shows the −log_10_(p-value) of the functional enrichment. (C) STRING network of the proteins contained in cluster 7.59, annotated as cytoplasmic translation (left). Exemplary HPA immunofluorescence microscopy images of proteins contained in the cluster showing the distinct staining pattern (right). (D and E) Representative images for the four different nuclear membrane-related clusters (D) and the four different microtubule-related clusters (E) displaying the different staining patterns in the respective clusters. (F) Staining of proteins from the uncharacterized microtubule clusters (E) in ciliated kidney cells (RPTEC/TERT1), confirming that these represent cilium-localizing proteins.

We applied the MAE-CellS-ProtS-Pool version of SubCell to represent all HPA images for the U-2 OS cell line (9,543 proteins) in an embedding space. Hierarchical Leiden clustering [33] of the embeddings at increasing levels of resolution (Fig. 5 A) enabled the construction of a hierarchical tree based on the embedding pattern similarities (Fig. 5B, Supplementary Table S3). The clusters were annotated using enrichment of Gene Ontology (GO) terms, which revealed cellular compartments and protein assemblies at different scales. Biologically meaningful clusters ranged from subcellular compartments at low resolutions to specific protein complexes at higher resolutions. For example, the cytosolic ribosome cluster 6.31 splits into subclusters 7.59, 7.74, and 7.78 (Fig. 5B). Protein-protein interaction data from STRING [34] validated that cluster 7.59 contained proteins that interact in the cytoplasmic ribosome (Fig. 5C). The concordance with known protein complexes exemplifies that SubCell detects subcellular compartments from light microscopy images beyond what humans can realistically annotate. We conclude that SubCell’s purely vision-based multiscale map can resolve individual large protein complexes.

#### Distinguishing clusters of dynamic and stable proteins

Another illustrative example of SubCell’s interpretative power is that the multi-scale map discerned two major clusters related to the nuclear envelope (6.34 and 6.22). The nuclear membrane cluster 6.34 contained two subclusters: Cluster 7.77 consisted of nuclear envelope proteins, and the unannotated Cluster 7.65 contained multiple nuclear lamina proteins (Fig. 5D, Supplementary Table S3). The other nuclear membrane cluster (6.22) was closely related to shuttling processes across the nuclear membrane: Subcluster 7.82 consisted of proteins driving mRNA processing, splicing, and export, whereas the proteins in the unannotated subcluster 7.36 were involved in nuclear-export and nuclear enzymatic function. Visually, the images in cluster 7.36 showed envelope staining and confined spots in the nucleus and cytoplasm. We hypothesize that these clusters contain proteins that are at different stages of transport. The separation of these clusters along the lines of biological function led us to conclude that the multiscale map built from SubCell embeddings could distinguish between dynamic (e.g. shuttling) proteins and stable (e.g. structural) proteins within subcellular compartments.

#### Vision-based multiscale cell map suggests new protein functions

A similar case to the nuclear envelope was observed for microtubule-localizing proteins. The map distinguished microtubule cytoskeleton staining (cluster 3.4) into two major classes (clusters 4.8 and 4.10). Cluster 4.8 consisted of spotty microtubule stainings, whereas the stainings in cluster 4.10 were continuous (Fig. 5B, E). The continuous pattern cluster represented common microtubule proteins and did not subdivide at higher levels of resolution (Fig. 5B, clusters 4.10, 5.17, 6.26, 7.52; Supplementary Table S3). Conversely, the spotty pattern class subdivided into a cluster related to cilia assembly and two clusters that were not enriched for a specific GO term (clusters 7.27, 7.57, and 7.85, Fig. 5B). The two unlabeled clusters contained some proteins implicated in cilia function (e.g., TTLL9 and NEK8 [35] in Cluster 7.57, WDR11 and CFAP54 [36]) in Cluster 7.85, Fig. 5B).

Although cilia have never been observed on U-2 OS cells, cilia proteins are known to be expressed in U-2 OS cells and to often localize to other cellular compartments in addition to cilia [37]. The staining pattern of Cluster 7.57 was consistent with pericentriolar material or centriolar satellites, known as transit stations for ciliary proteins and regulators of ciliogenesis [38, 39], whereas the spots of equal intensity across microtubules in Cluster 7.85 were suggestive of microtubule-mediated ciliary cargo transport. The Golgi-staining in Cluster 7.27 matched the trafficking route of ciliary membrane proteins, which are assembled at the Golgi and routed to cilia through vesicle-based transport [40] (Fig. 5E). In fact, several proteins in cluster 7.27 mediate protein transport to the cilia or are present in ciliary membranes, including ARL13B [41], IFT22 [42], OCRL [43], and PIK3R4 [44]. Conversely, Cluster 7.57 contained NEK8, known to engage in ciliary transport and biogenesis, as well as under-characterized ciliary proteins, such as TTLL9 and TEKT2 [45]. Cluster 7.85 contained additional, lesser-studied proteins, such as WDR11, a less-characterized protein that is ascribed to modulate ciliogenesis and ciliary signaling.

We hypothesized that, based on their pattern similarity with established cilialocalizing proteins, less characterized proteins in these clusters would localize to cilia in ciliated cells. We confirmed ciliary localization for established cilium proteins and new suggestions by staining ciliated cells with the same antibodies in a parallel running high-throughput study exploring the cilia proteome [37]. We confirmed the suggested proteins TAF1A (not previously linked to cilia), PIK3CD (previously implicated in ciliary functions but assumed to act outside of cilia), and CFAP54 (previously only reported in motile but not primary cilia), as well as the established cilium-localizing proteins TTLL9 and WDR11 in cilia (Fig. 5F), confirming SubCell’s pattern-based predictions.

We conclude that SubCell can detect protein functions from staining patterns, and thereby suggest new protein functions.

### SubCell provides novel opportunities for multimodal integration

Recent studies [46–48] have demonstrated that integrating biomolecular data across various modalities (gene, RNA, and protein levels) is crucial for a comprehensive understanding of cellular processes, defining the field of “functional genomics”. In this vein, we demonstrate that an image model like SubCell can inform a multimodal representation of a gene. We integrated SubCell embeddings of protein spatial distribution from HPA images and ESM2 [49], a well-established sequence-based model of evolutionary information, to create a unified embedding space that describes gene function across scales better than either modality individually.

#### Distinction between image- and sequence-based protein information

**Fig. 6:**
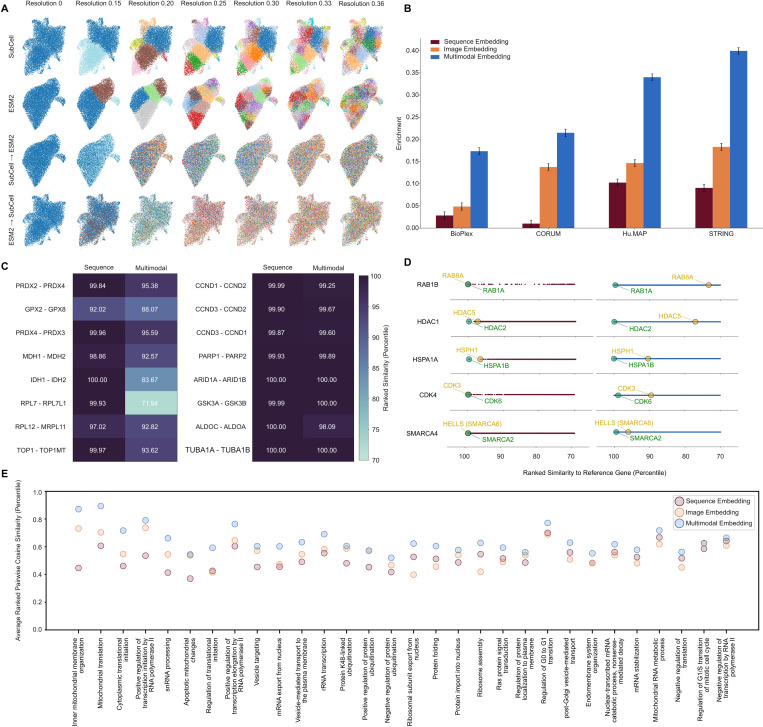
Evaluating the multimodal integration of image and sequence data. A) Top two: UMAPs of iterative Leiden subclustering of the SubCell MAE-CellS-ProtS-Pool embedding space and ESM2 embedding space, respectively, on proteins in HPA’s U-2 OS images; Bottom two: The same cluster assignments as directly above, but transferred onto the other embedding space. B) Similarity enrichment (calculated using the approximated Cliff Delta) of the sequence embedding (red), image embedding (orange), and multimodal embedding (blue) for interacting pairs as noted in the BioPlex, CORUM, Hu.MAP, and STRING databases. C) Heatmap tables displaying the similarity of embeddings for genes that are functionally divergent paralogs (left) and functionally redundant paralogs (right) in both the sequence and multimodal spaces. Here, similarity is quantified using the percentile of ranked similarity, with high percentiles signaling high similarity in an embedding space. D) Number lines displaying comparative differences in the similarity between functionally divergent paralogs (yellow) and functionally redundant paralogs (green) of a gene common between the two. The left group plots values in sequence space, and the right group plots values in multimodal space. E) Average similarity (again, percentile of ranked similarity) for gene members in selected location-dependent biological processes, across sequence (red), image (orange), and multimodal (blue) embedding spaces.

First, we established that the sequence and image data modalities capture different information about a gene. For visual confirmation, we assigned respective hierarchical cluster labels (as done for the multiscale map in the previous section) to both the SubCell and ESM2 embeddings using a common set of genes. We then transferred the cluster label of each gene from one embedding space to the other and visualized it with a UMAP. Clusters in SubCell did not translate to notable clusters in the ESM space, and vice-versa (Fig. 6A), suggesting that the two models capture largely orthogonal information. To quantify this, we plotted the standardized pairwise similarities of SubCell embeddings against those of the corresponding ESM embeddings and found no correlation (*R*^2^ of −2.00) (Fig. S4B). This analysis reveals a novel opportunity to create a more robust gene representation by combining embeddings.

#### Stronger interaction signals in a multimodal space

To create a multimodal embedding, we integrated the SubCell HPA and ESM2 protein embeddings using the MuSIC framework [32]. We evaluated these new embeddings on their ability to recover known interacting protein pairs documented in BioPlex [50], CORUM [51], huMAP [52], and STRING [34] databases using similarity enrichment. The multimodal embedding significantly outperformed each unimodal embedding on all tasks, and in a way that is more than additive (e.g., Cliff’s Delta of 0.09 for the sequence embedding, 0.18 for the image embedding, and 0.4 for the multimodal embedding on the STRING interaction task) (Fig. 6B). This suggests that a multimodal embedding can decode higher-order information between location (image) and sequence embeddings in a manner relevant to physical and biological interactions.

#### Detecting functional divergence of paralogs

We further explored whether the multimodal embedding contains deeper information about biological function than the ESM2-only embeddings, which are already known to have strong performance on function-based tasks [53]. We analyzed a sample of functionally divergent paralogs: genes that share a recent common ancestor but have evolved different roles within the cell. Using percentile ranking of pairwise similarities to compare across embedding spaces without explicitly normalizing them with respect to each other, we found that such paralogs were hardly separated in sequence space but were notably separated in multimodal space (Fig. 6C, right). This suggests that the multimodal space is organized to distinguish evolutionary divergence. To confirm that the organization of multimodal embeddings is not random, we also performed the same analysis for redundant paralogs, i.e., paralogs that retain the same function. These had high similarity in both the ESM2-only and multimodal embedding spaces, indicating that the multimodal embedding space still preserves strong functional relationships (Fig. 6C, left). For an explicitly comparative perspective, we selected genes with both functionally divergent and functionally redundant paralogs, observing similar trends in similarity (Fig. 6D). Using only the image embedding, functionally divergent paralogs were highly separated, but so were functionally redundant paralogs, indicating that images alone cannot describe such functional specificity (Fig. S4 C, D). Thus, the reorganization in the joint-embedding space more faithfully reflects gene function, likely because the multimodal embeddings add spatial context, and protein location is intrinsically tied to protein function and serves as a spatial axis for cellular evolution of specialized functions.

#### Biological process enrichment in an integrated embedding space

GO BP combines information on protein interactions and function, reflecting systems-level biological phenomena. We explored whether localization-specific BPs are more strongly enriched in the joint embedding space than either of the individual models, hypothesizing that this subset would benefit most from the integrated information.

We manually searched for localization-specific BPs associated with the same gene set used earlier in this section. Then, we calculated scores based on the average pairwise similarity ranking of the BP’s genes in each embedding space. The multimodal embeddings consistently show higher average similarity scores than the image and sequence embeddings alone for BPs that relate to localization or a specific cellular compartment (Fig. 6E). This is even true for temporally dynamic processes, such as the cell cycle. Comparative plots for the remaining 228 BPs enriched in the gene set (both localization-specific and non-localization-specific) can be found in Fig. S5. In total, these results suggest that the multimodal protein space has readily available information on systems-level cellular operations, positioning it as a useful tool for applications such as pathway discovery or functional annotation.

## 3 Discussion

We introduce SubCell, a suite of biological vision foundation models for encoding single-cell representations from fluorescence microscopy images, trained using a multitask, self-supervised learning framework. SubCell outperforms state-of-the-art models across a diverse range of tasks in single-cell biology, including localization prediction, cell phenotyping, morphological phenotyping, drug treatment prediction, and mechanism of action prediction. It readily generalizes to datasets with different cell types, channels, imaging modalities, and resolutions.

During the design and optimization of SubCell, we observed that typical learning frameworks, such as MAEs and vanilla contrastive learning-based methods, were unable to encode complex protein localization patterns as effectively as supervised methods. Introducing a protein-specific objective to maximize the similarity in representations between cells treated with the same antibody significantly boosted performance in protein localization prediction. To increase the applicability of our software suite to diverse image inputs, we trained SubCell with varying combinations of marker channels, broadly increasing the compatibility of SubCell with existing and future datasets.

We demonstrated that SubCell generalizes to unseen datasets acquired using different microscopy methods, novel cell lines, drug treatments, and even cells of other species, such as yeast. SubCell outperformed state-of-the-art models across these tasks without any finetuning. We demonstrated that our models predict cancer cell treatment effects more accurately than state-of-the-art models using the Bridge2AI-CM4AI dataset, thereby paving the way for improved quantification of protein relocalization upon perturbation. We further found that our models can serve as general, out-of-the-box feature extractors, as highlighted on the JUMP1 dataset. Remarkably, our models not only surpassed the performance of existing deep learning-based models trained on Cell Painting images in replicate retrieval tasks but also matched the performance for MoA prediction, all without finetuning. This underscores the versatility and robustness of our models, positioning them as essential tools for advancing high-throughput morphological analysis. Recent studies [4] have demonstrated that mislocalization of pathogenic coding variants commonly underlies human disorders, and that protein relocalization can open up novel therapeutic opportunities. We therefore anticipate that detecting and disentangling subtle spatial differences in subcellular protein localization across cells of varying morphology will become increasingly important in therapeutic research.

We hypothesize that SubCell performs well on a variety of tasks and datasets because it has learned the intricacies of subcellular architecture and cell morphology through exposure to nearly all intracellular human proteins across a wide range of cell lines. The novel proteome-guided training scheme using the HPA subcellular image dataset enabled SubCell to learn biologically meaningful attention patterns, thereby achieving exceptional generalizability to downstream tasks without the need for finetuning.

Biological images alone serve as the basis for the meaningful information that SubCell learns. This is demonstrated by the construction of the first vision-based multiscale map of subcellular protein architecture, which successfully categorizes subcellular protein localization by organellar structures and domains, as well as protein interaction and function. This map highlighted new avenues for utilizing microscopy data to decipher cellular phenotypes. It can be used as a vision-based data-driven alternative or supplement to GO for gene, protein, and pathway annotations. The annotations resulting from such analyses can help overcome the biases inherent in human-defined localization classes. Future comparisons of SubCell multiscale maps generated from genetically or drug-perturbed cells may reveal the rewiring of cellular organization or pathway interactions. We also envision that this map can be integrated into transcriptome-based models to provide improved biological context and facilitate causal reasoning.

Finally, we demonstrate that SubCell embeddings of protein cellular distributions are valuable for contextualizing protein sequence models. Integrating SubCell HPA embeddings of protein subcellular distribution with sequence embeddings led to superior performance on protein interaction and functional divergence tasks. Interestingly, while SubCell and ESM2 individually perform relatively poorly on these tasks, combining the two modalities improves performance, revealing biologically meaningful synergy. This extends to predicting biological processes, suggesting that spatial models are necessary to capture protein function beyond the molecular level and to approach a systems-level understanding.

SubCell is a generalizable foundation model that enables quantitative image representation for microscopy image analysis, independent of optimizing image analysis pipelines. To ensure SubCell is broadly usable by researchers worldwide, the model weights and source code are freely available and easily accessible. We provide tutorials, a ready-to-use application online, and an interactive version of the proteome-wide hierarchical cellular map. Beyond cell biology research, we envision SubCell to aid in drug and perturbation screening by providing more robust representations than human-crafted features, bringing us one step closer to virtual experimentation across data modalities and ultimately facilitating the construction of a universal virtual cell model.

## 4 Methods

### Datasets

We used the HPAv23 dataset to train SubCell and evaluated it using the other datasets. The descriptions of the datasets are as follows, and the details regarding their size and pixel resolution are provided in Supplementary Table S4.

### Human Protein Atlas Subcellular Dataset

The HPA subcellular dataset is a collection of immunofluorescence images encoding the expression and spatiotemporal distribution of 13,141 genes in 37 cell lines [5]. The cell lines represent a broad band of tissues, cell types, age, and sex: e.g., U2OS is an osteosarcoma cell line derived from the tibia of a 15-year-old female patient; ASCtelo is a primary mesenchymal stem cell line derived from the adipose tissue of a female donor; RPTEC/TERT1 is a primary kidney proximal tubular cell line derived from the kidney cortex of a male donor; SH-SY5Y is a neuroblastoma cell line derived from a bone metastasis in a 4-year old female patient; AF22 is an induced-pluripotent-stem-cell-derived neuroepithelial stem cell line from a 36-year old female patient; CACO-2 is a colon-derived adenocarcinoma cell line derived from a 72-year old male; U-251MG is a glioblastoma cell line derived from the parietal lob of the brain of a 75-year old male. The images were generated by staining the cells with DAPI, a fluorescent dye that labels DNA/nucleus, and antibodies that label the endoplasmic reticulum (ER), microtubules (MT), and the protein of interest (Protein). The protein localization in the dataset is divided into 35 organelles and fine subcellular structures. Version v23 of the image dataset was used. The single cells were extracted from the images using the segmentation masks generated using the recommended HPA-Cell-Segmentation method [24]. The segmentation masks were processed to remove nuclei touching the border and those that were too small, and then to merge the detections that were too close to each other. Altogether, 1,138,378 cells were extracted from the images and used from the dataset.

The dataset was split into training, validation, and test sets based on the antibodies, with an approximately 7:1:2 ratio. A multilabel stratification strategy [54, 55] was used to ensure a similar multilabel distribution between the sets. Further details on the data distribution among test, train, and validation can be found in the GitHub repository (https://github.com/CellProfiling/subcell-analysis).

### OpenCell Dataset

The OpenCell dataset comprises live-cell confocal images of 1,310 CRISPR-based, endogenously tagged proteins on the HEK293T cell line. The cells were imaged using a nuclear stain (Hoechst 33342) in conjunction with the protein. The single-cell images were generated by segmenting the nuclei and cropping a 256×256-pixel region around them. Overall, 94,426 cells were obtained. The annotations for protein localization are divided into 18 categories, including multi-localizing proteins.

### AllenCell Dataset

We used a subset of the Allen Institute Cell Explorer dataset [7] used in the DINO4Cells paper [16], containing 214,037 human induced pluripotent stem cells (hiPSC) from 25 isogenic cell lines, each containing one fluorescently tagged protein via CRISPR/Cas9 gene editing. The cells were imaged in 3D using fluorescently tagged markers for the nucleus and plasma membrane, as well as the protein. The masks for all the markers were provided within the dataset. For our analysis, we used the masked maximum projection of the channels across the z-plane. The cell cycle stage labels for the cells were annotated by experts and were used to validate the models.

### Yeast Dataset

This dataset contains fluorescence microscopy images of live Saccharomyces cerevisiae cells expressing a GFP-tagged protein of interest, along with two reference markers: RFP to label the cytoplasm and F-RFP to label both the nucleus and the bud neck via the septin protein CDC11 across 4,100 proteins [25]. Images were acquired using a high-throughput spinning-disc confocal microscope. Two datasets of single-cell image crops are provided: one for subcellular localization and another for cell cycle classification, both available at https://github.com/BooneAndrewsLab/CycleNET. Each dataset was manually annotated, comprising 22 localization classes and 9 cell cycle classes, respectively. Each dataset is split into training and testing sets. For our analyses, we excluded the “Over-segmented” and “Aberrant” categories from the cell cycle dataset, as these reflect segmentation artifacts or empty images rather than biologically meaningful states.

### U2OS FUCCI Dataset

The dataset comprises endogenously tagged cells expressing two fluorescent proteins, CDT1 and GMNN, which are fused to cell cycle regulators, allowing for the monitoring of the cell cycle [1]. Along with these proteins, the cells were also tagged with a protein of interest and a microtubule marker. The cell segmentation was done using the HPA-Cell-Segmentation method [24]. Overall, 357,083 cells were obtained, for a total of 1,166 antibodies. A polar coordinate pseudotime model was employed to generate a continuous representation of the cell cycle position, spanning from 0 to 1. Furthermore, based on the cell cycle estimation, the cells were labeled between three discrete stages: G1, G1S, and G2.

### Dataset of drug-treated triple-negative breast cancer cells

The image dataset of drug-treated triple-negative breast cancer cells originates from the Cell Maps for Artificial Intelligence (CM4AI) project, a component of the U.S. National Institute of Health’s (NIH) Functional Genomics Data Generation Project within the Bridge2AI program [29]. We used only the subcellular proteomics, i.e., immunofluorescence imaging dataset, and of this, only the dataset focusing on the triple-negative breast cancer cell line MDA-MB-468. The dataset comprises 63x confocal images of cultured MDA-MB-468 cells treated with the drug Paclitaxel (NSC 125935, Selleckchem S1150), the drug Vorinostat (SAHA, Selleckchem S1047), or untreated. The experimental workflow followed the workflow applied in the Human Protein Atlas (HPA) subcellular section: The cells were seeded in fibronectin-coated glass-bottom 96-well plates (18,000 cells per well), treated with the different treatment conditions, incubated for 24 hours, fixed with 4% paraformaldehyde (PFA) and stained with a nucleus marker (DAPI), a microtubule marker (alpha-Tubulin antibody, Abcam, RRID: AB 2241126), an ER marker (Calreticulin antibody, Abcam, ab2908, RRID: AB 303403), and an antibody against a protein of interest from the HPA antibody library. Each well was stained with a different antibody against a protein of interest. For each protein of interest, the experiment for the different treatments and the staining were done simultaneously to minimize effects unrelated to the perturbation. The dataset contained the images acquired for 454 different proteins.

### JUMP1 Dataset

A subset of the JUMP1 pilot dataset (cpg0000-jump-pilot) was used [9]. The complete JUMP-Pilot dataset contains 300 million images of U20S and A549 cells under both chemical and genetic perturbations at both short and long time points. JUMP1 data, segmentation masks, metadata, and CellProfiler profiles are publicly available at https://cellpainting-gallery.s3.amazonaws.com/index.html#cpg0000-jump-pilot/. A subset of plates was selected by filtering for chemical perturbations, U2OS cells, and the absence of any antibacterial agent. After filtering eight plates remained, BR00117010, BR00117011, BR00110712, BR00117013, BR00117024, BR00117025, BR00110726 and BR00116995. BR00116995 contained an unusual number of empty wells and was excluded from our analysis. The plates BR00117010–13 were imaged at 24 hours, and plates BR00117024–26 were imaged at 48 hours. The layout of treatments across wells was the same for each plate, containing 64 negative control wells and 320 wells with chemical perturbation from one of 302 compounds. We used 77 pre-defined MoA classes [56]. When calculating MoA mAP, and NN-acc, only 192 compounds were used because they shared at least one MoA with another compound.

### Vision Transformers

Vision transformers (ViTs) are based on the transformer architecture [? ], originally developed for textual data [57], which utilizes a novel self-attention mechanism. A set of word tokens is fed into multiple attention heads, which convert them into keys, queries, and values to get an attention matrix. In ViT architecture, the input tokens are created by dividing the image into fixed-sized patches similar to the words in a sentence. The transformer architecture effectively captures global relationships across the image without relying on convolutional architectures. We selected the ViT-B model, which has ˜86 million parameters and a patch size of 16×16 pixels, for our experiments. We further trained a ViT-B model with a patch size of 8 to obtain finer-grained representations, and the results are presented in Supplementary Table S5. We also trained a ViT-L model (307 million parameters) and showed the result in Supplementary Table S6.

### Masked autoencoders

Masked autoencoders (MAEs) [18, 58] introduced an efficient way to train vision transformers at scale in an unsupervised manner. A random subset of the tokens in an image is masked out, and the remaining tokens are fed into the encoder. After encoding, the masked tokens are reintroduced, and all the tokens are fed into a small decoder that reconstructs the original image from the tokens. A reconstruction loss is applied to the masked patches to facilitate learning. After the pre-training, the encoder can be used as a feature extractor for the downstream tasks, and the decoder is discarded. A detailed architecture description is shown in Fig. 1. Since only the non-masked tokens are processed through the encoder, MAEs require less computation than supervised training. The tokens to be removed from the image are chosen randomly. However, since we also had the cell masks available, we explored mask-aware token removal, i.e., removing the tokens present in the cells (Supplementary Fig. S6), and the results are shown in Supplementary Table S7.

### Contrastive learning

We employed contrastive loss to train the models with cell-specific and protein-specific objectives. Contrastive learning is a self-supervised learning paradigm that focuses on learning image representations by focusing on the similarities and dissimilarities between data points [59]. The loss aims to minimize the distance between the positive pairs and maximize the distance between the negative pairs in the representation space. For the cell-specific objective, the positive pairs in a batch are generated by augmenting the cell image with different augmentations, and the rest of the batch is treated as negative pairs. For the protein-specific objective, all cells treated with the same antibody are considered positive samples, and the rest are considered negative samples.

### Attention-based Pooling

Attention-based pooling [60] summarizes the contribution of a feature in a large feature set to form a single feature vector, where each feature in the set is weighed based on its importance. Typically used for multi-instance learning, it provides an explainable way to interpret the importance of each feature. Instead of computing the mean of the token embeddings, we used attention-based pooling weights to get a single feature embedding for the image. Each attention head processes the tokens separately and outputs an aggregated feature vector. We empirically chose the number of pooled attention heads to 2 for our experiments, and concatenated the outputs to double the output feature dimension from the encoder. We observed improved performance when using the pooling module compared to simple mean pooling. Further, the module also provides an interpretability measure in the form of a token importance map.

### Multi-task Learning

A multi-task framework was used to learn a diverse set of features for the ViT encoder. To enable effective contrastive learning with the MAE framework, only one of the views is fed to the decoder, similar to [61, 62], and the augmentation strategy was modified. The images were augmented with geometric transformations for the view being fed into the decoder, as MAEs reconstruct pixel intensities. Meanwhile, the other view was augmented with both geometric and color transformations. The output of both views is passed through the attention pooling module, and the contrastive loss is computed based on the feature representation. For the ViT-ProtS-Pool model, both views were augmented with geometric and color transformations.

The geometric augmentations used in our experiments were random vertical and horizontal flips, random affine with rotation of 90 degrees, translation of 0.2, and scale factor (0.8, 1.2), and random perspective transform with a distortion scale of 0.25. For the color transformations, we used per-channel transforms as used in Doron et al. [16], with random brightness and contrast scaling of 0.5, a Gaussian blur with a kernel size of 7 and sigma range (0.1, 2.0), and adjusting sharpness with a factor of 2, and random erasing the parts of the channels. We also randomly erased channels with a probability of 0.25 if the experiments were performed on more than three channels. We used intensity rescaling of the protein channel with a probability of 0.25. We randomly masked the cell crops in a batch with a probability of 0.5 to guide the model in ignoring surrounding cell regions present in the crop. This reduces the need for cell segmentation in the analysis, allowing the model to be applied directly to the raw image in a grid-like manner for feature extraction. Furthermore, the augmentation focuses attention on regions within the cell, rather than on the background.

### Model Training and Inference

All models were trained for 300 epochs using the AdamW optimizer [63] with a base learning rate of 1e-4 and weight decay of 0.05. We used a linear warmup for the first five epochs and decayed the learning rate using a linear decay schedule without restarts. The training batch was modified to ensure an equal distribution of cells from different antibodies and was empirically set to 8 cells per antibody. For training, a 896×896 pixel region around the cell was extracted and resized to 448×448 pixels to be fed into the models.

We conducted an ablation study to determine the optimal cell crop size for inference (shown in Supplementary Fig. S7). We found that the models performed best when using crops of 640×640 pixels centered around the cell. The inference was performed on cell crops using cell masks to ensure a fair comparison with other methods and to remove the effects of surrounding cell regions.

### Analysis of the multi-task components of the model

We initiated experiments with masked autoencoders (MAEs) using different mask ratios (Supplementary Table S7). With available cell masks, we introduced a masking strategy guided by these masks. We used cell masks to direct the masking strategy rather than randomly removing patches in the image for reconstruction (Supplementary Fig. S6). As shown in the results (Supplementary Table S7), increasing the cell masking ratio while keeping the overall masking ratio constant improved model performance in both tasks. However, we observed that while a higher masking ratio enhances performance in the cell-line classification task, it results in poorer performance in protein localization prediction tasks. Overall, we found that the MAE models fall behind the DINO model in protein localization but are closer in the cell-line classification task. We hypothesize that since MAEs focus on the structure of cells in the images and protein localization in HPA is independent of cellular structure, when the cells are unperturbed, the MAEs perform poorly in this task.

Subsequently, we trained the models with cell-specific contrastive loss and its combination with the MAEs (Supplementary Table S8). The models trained with MAE and contrastive loss outperformed those trained with just contrastive loss for cell-line classification; however, they lag in protein localization prediction tasks. The models outperformed the models trained with just MAE in both tasks. We observed that increasing the masking ratio negatively impacts the models’ localization performance, and the cell masking strategy further deteriorates performance. This might be attributed to differences in the images the encoder sees, i.e., in one view, certain parts of the cell are masked, for example, the regions with protein, while in others, they are not. Still, the best model could not match the performance of the DINO model.

We performed experiments using the protein-specific contrastive loss framework (Supplementary Table S9). We evaluated the addition of protein-specific loss in the base model, i.e., ViT, and its addition to the different top-performing cell-specific and MAE configurations. We found that the model trained with just the protein-specific loss (ViT-ProtS) performed best for the protein localization task (micro AP of 0.878), outperforming the DINO (0.863), weakly-supervised (0.869), and bestfitting (0.83) models on the HPA test set, while the model with the MAE, cell-specific, and protein-specific loss (MAE-MR0.25-CMR-0.0-CellS-ProtS) performed second best (0.872). The detailed architecture of the multitask model is shown in Supplementary Fig. S1. The ViT model trained with cell-specific and protein-specific loss performed best on the Kaggle test set for protein localization but lagged behind in cell-line classification.

Finally, we conducted experiments to evaluate the utility of the pooling module on the two best-performing models from the previous experiments (Supplementary Table S10). The pooling module improved the performance of both models for both tasks in the HPA dataset. A significant improvement was observed in the ViT-ProtS model for the cell-line classification task, with the model’s performance increasing by 0.017%, matching that of the MAE model.

### MLP classifier

For the supervised learning tasks, i.e., localization prediction, cell cycle stage prediction, we trained a multi-layer perceptron (MLP) classifier on the features from the models and generated the predictions. We used the same three-layer classifier architecture as Doron et al. [16] and trained the classifiers using the focal loss to address the class imbalance in the dataset. The classifiers were trained on the features extracted from the same images used to train and validate the encoders. We trained the models for 200 epochs using the AdamW optimizer with a learning rate of 1e-3, and reduced the learning rate by 0.5 if the validation mAP didn’t improve for more than four epochs. The training is stopped if no improvement is observed for 20 consecutive epochs. We trained ten classifiers with different seeds for each task and reported the mean and standard deviation of the metrics.

### Evaluation on HPA datasets

We trained two sets of classifiers for the localization classification task. The first set comprised the 19 categories specified in the Kaggle challenge, while the second set encompassed a broader range of 31 categories. This approach enabled us to perform a fair comparison with the bestfitting and DINO4Cells-HPA models, further demonstrating our models’ capability to encode more comprehensive localization information compared to previous methods. The results of the classifier trained with 31 classes are shown in Supplementary Table S11. We evaluated the performance of the models at the field of view (FOV) level on the HPA test set and on the single-cell level on the hidden test set of the Kaggle single-cell classification challenge. We averaged the predictions of cells in an FOV to report the FOV-level classification results. We reported micro and macro average precision (AP) as the classification metrics and used multilabel ranking average precision (MLRAP) and coverage error to evaluate the multilabel performance on the test sets. MLRAP evaluates how well a model ranks predicted labels by measuring the proportion of relevant labels ranked higher than irrelevant ones, while coverage error computes the average number of labels to be included in the prediction such that all true labels are predicted.

### Evaluation on the OpenCell dataset

The cell features were extracted using the DNA-Protein variant of SubCell. We evaluated the features on the original resolution images and the resized images, which were matched to the pixel size of HPA images cropped to 640×640 pixels, and found that the features extracted by resizing performed better. We filter out the multi-localizing proteins and aggregate the single-cell features to create FOV-level profiles in the dataset. We employed the K-Means clustering algorithm for unsupervised clustering of the features and evaluated the agreement between the resulting clusters and the OpenCell annotation labels to compare the feature representations across the models. We reduced the number of features by keeping the top 100 principal components to minimize the noise in clustering. The number of clusters was set to the number of localization categories in the data. The clustering labels were then compared with the ground truth labels using the adjusted Rand Index, which assesses the similarity in cluster assignments, and V-Measure, which assesses the homogeneity and completeness of the assignments.

### Evaluation on the AllenCell dataset

The cell features were extracted using the DNA-Protein and MT-DNA-Protein variants of SubCell. Different channel combinations and image normalization techniques were evaluated (Supplementary Table S1). The images were resized to match HPA pixel size and cropped to 640×640 pixels for inference. We used the same training and test sets as those in the DINO4Cells paper [16]. Additionally, a validation set was extracted from the training set to choose the optimal model weights during training. Ten different classifiers were trained with different random initializations of the model weights, and the mean and standard deviations were reported.

### Evaluation on the FUCCI dataset

The cell features were extracted using the MT-DNA-Protein variants of SubCell. The images were resized to match HPA pixel size and cropped to 640×640 pixels for inference. Since the DNA channel is not present in the images, three different combinations of the CDT1 and GMNN channels were used to substitute for the channel in the experiments, i.e., passing an empty image, passing a nuclear mask with a constant value, and passing the nuclear channel as the average of the cell cycle markers (CDT1 and GMNN). The nuclear mask was obtained by simply performing Otsu’s thresholding on the mean image of CDT1 and GMNN channels. The dataset was split into 10 folds stratified by the antibody ID, and cross-validation performance was recorded. For each fold, models were trained 10 times each with different random seeds to account for the randomness in the predictions. For the regression task, i.e., predicting pseudotime given cell features, we used deep evidential regression training [64], which learns to account for both aleatoric and epistemic uncertainties in the prediction.

### Evaluation on the yeast dataset

Using the provided training and test sets, we trained each model with 10 different random seeds and averaged the resulting performance metrics and confusion matrices across seeds. We retrained the original CycleNET and DeepLoc [25] models on the cell cycle classification and subcellular localization tasks, respectively. For SubCell, we first preprocessed the image data by applying global min-max normalization and resizing the image crops to 1×, 2×, 3×, or 4× their original dimensions. We then generated feature embeddings using the two best-performing models. To achieve this, we mapped the imaging channels as follows: the nucleus and bud neck channels were input into the SubCell nucleus channel, the cytoplasm channel into the SubCell microtubule channel, and the GFP-tagged protein channel into the SubCell protein channel. We trained both two-layer MLPs and logistic regression classifiers using these SubCell embeddings as input features. For comparison, we also trained classifiers directly on globally min-max normalized pixel values. MLPs were trained for up to 100 epochs, with the best-performing model selected based on test set accuracy. All reported results are computed on the provided test set.

### Analysis of the factors of variation and subsequent integration

As shown in Doron et al. [16], self-supervised features often encode diverse factors of variation that are learned during training. Since the HPA dataset was acquired over more than a decade using different microscopes and experimental setups, we found that SubCell features also encode several technical variations, namely, variations across cell lines, microscopes, and plates (See Supplementary Fig. S8). We utilized Harmony [65], an algorithm designed for gene-expression data, to integrate across these factors of variation and remove confounding effects. Specifically, we integrate the features across cell lines, plate IDs, and the microscopes.

We employed a mutual information-based metric [66], following the same procedure as Doron et al. [16], to investigate the hierarchy of features in relation to the factors of variation, namely protein localization, cell lines, and well IDs. We first calculate a discrete joint distribution of a factor of variation. The number of bins for the distribution was set to 50. Next, the mutual information between the factors of variation and individual features was calculated, estimating how informative the feature is to each factor of variation. Finally, the features were ranked based on the factor of variation for which they’re more informative. Finally, the percentage of features that are most informative for each factor of variation was calculated and shown in Fig. S4C.

For the subsequent biological analysis, we performed the analysis with different harmonized features and presented the best results.

### Aggregating profiles over cell populations

We followed the same methodology as Doron et al. [16] to create the similarity matrices of cell lines and protein localizations. We averaged all the single-cell features in the populations and reduced the dimensionality of the features to keep the top ten principal components. We removed the cell lines and localization categories that were not present in the bulk RNA-seq data and the protein hierarchy, respectively. Comparison against other models was assessed using the Mantel statistic. [67]

### Evaluating attention maps

We quantified the regions our models attend to in a cell by calculating the correlation and Intersection over Union (IoU) between the attention maps and the input channels. For the attention analysis, we used the output of the attention head in the last layer of the ViT model with respect to the [CLS] token and the pooled attention heads. For correlation evaluation between input channels and attention maps, we normalize each input channel and attention output to the range of 0 to 1. The correlation was calculated for each input channel and the attention map, resulting in a 14 × 4 = 56-dimensional feature vector for each cell. For the hierarchy analysis, only the protein channel was used. To perform the UMAP visualization of the correlation profiles, we aggregated the profiles over the FOVs. The results for the ViT-ProtS-Pool model are shown in Supplementary Fig. S3.

### Evaluating protein relations across known biology

The single-cell features were combined across proteins to create the protein representation. We utilized the Reactome [27], Gene Ontology (GO) [28] cellular compartment (CC), and biological process (BP) databases for our analysis. The relationships between proteins were used to establish the hierarchy among different groups in the database. The top two levels of this hierarchy were used in our evaluation. Protein groups with fewer than ten members were excluded, and proteins present in more than five groups were also removed. For GO:BP level 1, 8208 proteins across 63 groups were analyzed, while at level 2, 5895 proteins across 108 groups were included. For GO:CC level 1, 8784 proteins in 62 groups were used, and at level 2, 8734 proteins in 86 groups were utilized. For Reactome, level 1 included 6692 proteins in 28 groups, and level 2 included 6382 proteins in 131 groups. The datasets were divided into different training and validation sets for 10-fold cross-validation. We perform a hyperparameter search to find the optimal parameters resulting in the best performance for each configuration. The hyperparameters that were optimized were the number of layers: {1,2,3}, hidden MLP dimensions: {128,256,512}, and dropout: {0.0,0.1,0.2,0.3,0.4,0.5,0.6,0.7,0.8}.

### Drug Prediction on the Bridge2AI dataset

The dataset was divided into three treatment categories: Paclitaxel, untreated, and Vorinostat. We perform a ten-fold cross-validation for the drug prediction task on the Bridge2AI dataset by dividing it into training and validation sets, stratified by protein. The classifiers were trained on the cell features obtained from the models. For each fold, ten classifiers were trained using the same hyperparameter search as the previous section. Finally, the results from all the folds were assembled, and final classification metrics were computed.

### JUMP Evaluation

Images were processed for both DeepProfiler and DINO4Cells-CP as described in their respective publications. The DeepProfiler package “prepare” and “export-sc” commands were used to obtain illumination-corrected and normalized single-cell crops. For performing inference with our models, single-cell crops were min-maxed, normalized, and then upscaled to match the physical pixel size of HPA images. Raw single-cell profiles underwent several standard post-processing steps before being used in downstream analysis. First, single-cell profiles are combined with FOV profiles by either mean or median aggregation. Second, FOVs are mean-aggregated to well-level profiles. Feature selection is optionally applied to well-level profiles by variance and feature correlation thresholding. Next, profiles underwent two rounds of normalization. 1) Z-score or MAD-robustize standardization was used to center the well-level profiles of each plate. 2) Sphering with respect to negative control wells across all plates was used to account for plate-level batch effects. Both ZCA and PCA sphering transforms were tested. All models were tested with all combinations of post-processing steps. This included switching the order of normalization steps and omitting sphering altogether. Feature selection, standardization, and sphering were performed with the pycytominer [68] package.

Well-level profiles were used to evaluate replicate retrieval. To evaluate MoA prediction, well-level profiles from the same compound treatment were mean-aggregated across plates to create a “consensus” profile for each compound. Mean average precision (mAP) and nearest neighbor accuracy (NN-acc) were measured using the cosine similarity of well-level and consensus profiles for replicate retrieval and MoA identification, respectively. Max-interpolation was applied to every precision-recall curve before computing mAP (see Supplementary Methods). Note that many compounds were annotated with multiple MoAs. Two consensus profiles were considered a match if they shared at least one MoA label.

Additionally, already-processed CellProfiler features were tested. They were made available with the JUMP1 data and had undergone MAD-robustization to the median of negative control wells per plate and feature selection by variance and correlation thresholding. Results for these profiles were not as high-performing as the CellProfiler features we processed and were thus not reported in the benchmark.

### Vision-based hierarchical organization of the human proteome

The HPA dataset was embedded using the MAE-CellS-ProtS-Pool and ViT-ProtS-Pool, respectively. Further processing and analysis of the embeddings was conducted in Python 3.10.14 using ScanPy 1.10.2. To remove unwanted sources of variance from the HPA protein single-cell embeddings, the embeddings were normalized for the covariates plate ID and microscope using the PyTorch implementation of the Harmony algorithm [65].

For this analysis, we focused on the cell line with the highest number of stained proteins, U2OS. The single-cell image embeddings were aggregated into one average embedding vector for each protein. For this, all antibodies with a reliability rating of ”uncertain” were removed, as well as those that stained more than one protein. Additionally, if there were stains from multiple antibodies for a protein, the most reliable antibody was selected based on the HPA antibody reliability score if the antibodies had different ratings; otherwise, one antibody was chosen randomly.

The resulting protein embedding, consisting of 9386 proteins, was (sub)clustered at multiple resolutions (0, 0.15, 0.2, 0.25, 0.3, 0.33, and 0.36) using the Leiden algorithm [33] with decreasing numbers of neighbors in the corresponding neighborhood graph (125, 100, 90, 55, 40, 25, 10) to focus on the global structure at lower resolutions and local structure at higher resolutions. For this, all proteins were clustered at a resolution of 0; the resulting clusters were then subclustered at a resolution of 0.15, and so on. From this, a hierarchical graph was constructed, where a node was created for each cluster at each hierarchy level, and edges were drawn between the nodes to indicate which clusters from a lower resolution yielded which clusters at the next higher resolution.

Additionally, a functional enrichment analysis was performed for each cluster using the G:Profiler Python package v1.0.0 [69]. The Gene Ontology (GO) Cellular Component ontology was used for resolutions 0, 0.15, 0.2, 0.25, 0.3, and 0.33, and GO Biological Processes and GO Molecular Functions ontologies were used for the highest resolution. The human genome was set as background, the g:SCS algorithm was used to compute multiple testing corrections for p-values, and a significance threshold of *p* < 0.05 was selected. Each node in the hierarchy graph was labeled with the most significantly enriched term.

### Establishing differences between ESM2 and SubCell embedding spaces

To investigate the orthogonality between sequence and image embeddings, we first created a set of image embeddings using the MAE-CellS-ProtS-Pool model and performed Leiden clustering in the same way as described in the previous section, using the same gene set. Then, we used the ESM2–650M model [49] to embed genes with evolutionary information derived from their sequences. We used the canonical UniProt sequence for each gene. We then assigned the cluster labels from the image embedding’s clustering onto the genes in ESM2 space. We created 2D UMAPs as a rough visualization of how well the clusters transferred from the SubCell image space to the ESM sequence space. No apparent clusters appeared for the ESM embeddings, especially at higher resolutions. The same process was repeated, this time clustering the ESM2 embeddings of the genes and then transferring the cluster labels to the SubCell embeddings. This yielded the same outcome. To quantify these differences, for each embedding space, we Z-score normalized each of the embeddings and calculated all pairwise cosine similarities in a matrix. Then, we plotted the datapoints such that the x-dimension represents the pairwise cosine similarity between a gene pair in the image space, and the y-dimension represents the similarity between the same gene pair in the sequence space. If ESM2 and SubCell created embeddings that shared the exact same information, we would expect the data to have an *R*^2^ value of 1. If they inform each other worse than just predicting using the average of the data points, the *R*^2^ would be negative.

### Multimodal Integration

We used the unsupervised MuSIC [32] coembedding scheme to integrate the protein image embeddings with protein sequence embeddings. We maintained the same hyperparameters and training settings as those used in the original work.

### Assessing enrichment of interacting pairs in the multimodal space

To assess whether the multimodal embedding encodes relevant biological information, we constructed a similarity enrichment evaluation task, employing a strategy similar to that of Schaffer et al. We used the CORUM [51] database.MAP [52], BioPlex [50], and STRING [34] databases for the analysis. For the CORUM dataset, we used 2,708 proteins with 88,288 interactions, for the hu.MAP dataset, we used 6,334 proteins with 33,245 interactions. For the BioPlex dataset, we used 8,593 proteins with 54,748 interactions, and for the STRING database, we used 8,956 proteins with 107,597 interactions. We first calculate the similarities between the protein embeddings and divide the databases based on interactions. We then calculate the Cliff’s Delta for the similarity between the interacting and non-interacting sets of proteins. We repeatedly sample 10,000 interacting and non-interacting protein pairs a thousand times to establish statistical significance.

We performed this analysis comparatively across different image models (DINO4Cells-HPA, bestfitting, SubCell ViT-ProtS-Pool, SubCell MAE-CellS-ProtS-Pool). In that analysis, we included an additional multimodal integration method (MIRAGE [48]) to compare different co-embedding methods.

### Detecting functionally divergent paralogs

To find examples of functionally divergent paralogs, we manually searched UniProt using the “Paralogs” section, which is contained in most entries. Knowing that true functional divergence is more likely to occur with subcellular localization divergence, we selected these paralogs by cross-referencing with the subcellular localization information provided with each entry. To find examples of functionally redundant paralogs, we again manually searched Uniprot, looking for paralogs with high sequence similarity. The distribution of pairwise similarities in the multimodal space vs. the ESM2 space is different, meaning that we cannot directly compare the embeddings of genes in each space. Instead, for a fair comparison, we ranked the pairwise cosine similarities of all genes in each space and calculated a percentile, representing the importance of each pairwise relationship relative to the rest of the relationships in the embedding space.

For each (functionally divergent and redundant) paralog pair, the ranked pairwise similarities were computed in each space and compared. In Fig. 6D, this comparison is displayed using a heatmap. In 6E, it is displayed using number lines for a set of genes that have both types of paralogs.

### Multimodal enrichment for biological processes

To select biological processes for evaluation, we used the entire gene set for which we had embeddings (i.e., the gene set for which we had images to create a SubCell embedding) and performed Gene Ontology Biological Process enrichment using g-Profiler. The results contained Biological Processes (BP) that were statistically enriched within our gene set. Since Gene Ontology is a tree-based annotation system, we removed any BPs that were parents of other BPs in our list of results. We also removed any BPs that were clearly cell-type specific (e.g., Myoblast differentiation). We then selected 30 of these that are associated with subcellular localization (e.g., post-Golgi vesicle-mediated transport) and/or have sister processes that occur in other compartments (e.g., mitochondrial translation). For each BP, we calculated all pairwise ranked similarity percentiles (as described above) of the participating genes in an embedding space and averaged them. These averages were calculated and plotted for all BPs across the image, sequence, and multimodal embedding spaces.

## Supplementary Material

Supplement 1

## Data Availability

The model and data are available at: https://virtualcellmodels.cziscience.com/. The HPA dataset is available in the Human Protein Atlas database (www.proteinatlas.org). The cropped image dataset used for training the models is available in the S3 bucket: s3://czi-subcell-public/
