# Supplementary material for: SubCell: Proteome-aware vision foundation models for microscopy capture single-cell biology
Source: bioRxiv. 2025 Oct 30:2024.12.06.627299. Preprint. [Version 2] doi: 10.1101/2024.12.06.627299 (PMC12636579; doi:10.1101/2024.12.06.627299)
Supplement: Supplement 1 [file media-1.pdf]

SubCell: Proteome-aware vision foundation models for microscopy capture single-cell biology

Ankit Gupta[1], Zoe Wefers[2,3], Konstantin Kahnert[2], Jan N. Hansen[1,2], Mohini K. Misra[2], Will Leineweber[2], Anthony Cesnik[2], Dan Lu[4], Ulrika Axelsson [1], Frederic Ballllosera[2], Russ B. Altman[2], Theofanis Karaletsos[4], Emma Lundberg[1,2,5,6*]

[1] Science for Life Laboratory, School of Engineering Sciences in Chemistry, Biotechnology and Health, KTH Royal Institute of Technology, Stockholm, Sweden.
[2] Computer Science Department, Stanford University, Stanford, CA, USA.
[3] Department of Bioengineering, Stanford University, Stanford, CA, USA.
[4] Chan Zuckerberg Initiative, Redwood City, CA, USA.
[5] Pathology Department, Stanford University, Stanford, CA, USA.
[6] Department of Applied Physics, Stanford University, Stanford, CA, USA.
[7] Chan Zuckerberg Biohub, San Francisco, CA, USA.

*Corresponding author(s). E-mail(s): emmalu@stanford.edu;
Contributing authors: ankit.gupta@scilifelab.se; zwefers@stanford.edu; kkahnert@stanford.edu; jnhansen@stanford.edu; mmisra@stanford.edu; wleinewe@stanford.edu; cesnik@stanford.edu; dlu@chanzuckerberg.com; dlu@chanzuckerberg.com; fredbn@stanford.edu; russ.Altman@stanford.edu; tkaraletsos@chanzuckerberg.com;

1 Supplementary Methods

Evaluating the performance of the models on a broader range of localization categories

We also trained classifiers on the features extracted from the models to evaluate the performance of the models on the broader range of categories present in the HPA dataset (see Table S1, Original annotation). The results, shown in Table S7, reveal similar performance of our models as compared to the challenge categories.

Choosing the input size for inference

We performed experiments to determine the optimal resolution to perform inference for the models on the HPAv23 test set, Figure S3. We found that results vary across different metrics, but ultimately decided that the crop size of 640x640 pixels with any resizing resulted in decent performance across all metrics for both cell-line classification and protein localization prediction, and used it to evaluate the models.

Training finer-grained and larger models

We also experimented with a smaller patch size to capture finer-grained features in the images and evaluate if the performance of the models could be further improved. We conducted experiments using the ViT-ProtS-Pool model with various combinations of marker channels. As shown in Table S8, we found that decreasing the patch size to 8 improved the performance of the models with fewer marker channels; however, it didn't surpass the performance with the patch size of 16 when all the marker channels were present for both localization prediction and cell-line classification tasks. We further trained a ViT-L model with 300M parameters (Table S9) and found that the ViT-L model improved the localization prediction performance. However, a trend similar to decreasing the patch size was observed for the cell-line classification task.

Evaluating BYOL as the protein-specific loss

We also explored the utility of another self-supervised method, i.e., a self-distillation approach named BYOL, for the protein-specific loss, and compared it with the contrastive approach. In our experiments (Table S 10), we found that BYOL did not outperform contrastive learning in any of the multi-task settings.

JUMP technical effects visualization

We perform a UMAP visualization of the post-processed embeddings from all models. We used the UMAP algorithm with correlation as the distance metric and set the number of nearest neighbors to 50 and the minimum distance between points to 0.7. Plate-batch effects appear to be more pronounced in deep learning models as compared to CellProfiler (Figure S6). This is in accordance with findings from other studies (20). Additionally, we visualize UMAPs colored by well-position and qualitatively observe mild well-position effects in all models (Figure S 7). Lastly, we observe

interesting patterns in the separation of control and perturbed profiles in the UMAP space (Figure S8). We note that the models whose optimal post-processing pipeline performed sphering before centering showed significant separation, while other models did not. Thus, we hypothesize that sharp separation is likely an artifact of the order of normalization steps. While the ideal batch correction methods for CellProfiler features have been studied extensively, less is known about the best post-processing pipeline for deep-learned profiles (6). We highlight this as an interesting area for future investigation.

Perturbation morphological profiling metrics

To compute mean Average Precision (mAP), we go through all well profiles for replicate retrieval and each consensus profile for the mechanism of action (MoA) identification. For each profile, we treat it as a query profile and compare it against all other profiles. Average precision is calculated by taking the area under the precision-recall curve for a given query compound. Let f_i be a query profile and $\{f_1, f_2, \ldots, f_n\}$ be all other profiles. Let y_i and $\{y_1, y_2, \ldots, y_n\}$ be the corresponding labels, which in our case are either compounds or MoAs. Assume $\{f_1, f_2, \ldots, f_n\}$ are ordered by increasing cosine distance to f_i. The precision and recall values are calculated as follows.

$$P_j = \frac{\sum_{k=1}^{j} I_{(y_k = y_i)}}{j} \tag{1}$$

$$R_j = \frac{\sum_{k=1}^{j} I_{(y_k = y_i)}}{\sum_{k=1}^{n} I_{(y_k = y_i)}} \tag{2}$$

The standard precision-recall curve would be defined by $[R_1, R_2, \ldots, R_n]$ and $[P_1, P_2, \ldots, P_n]$. However, not all compounds and MoAs are equally represented in our dataset. Some query profiles have many more matching profiles to recall than others. To account for this, we max-interpolate the precision-recall curve for each query profile before computing the average precision. For all queries, we use the same recall axis based on the total number of profiles, $\left[0, \frac{1}{n}, \frac{2}{n}, \ldots, \frac{n-1}{n}, 1\right]$, and we define an interpolated precision function defined below. Lastly, we compute its average over the new fixed recall axis.

$$P_{\text{inter}}(r) = \max\{P_k : R_k \geq r\} \tag{3}$$

$$\text{MaxInter-mAP} = \frac{1}{n} \sum_{k=0}^{n} P_{\text{inter}}\left(\frac{k}{n}\right) \tag{4}$$

Nearest Neighbor Accuracy is the fraction of profiles whose nearest neighbor by cosine distance shares the same compound, or MoA, annotation. When we calculated nearest neighbor accuracy, we excluded profiles from the same plate as the query profile.

The random baseline was computed by generating a random symmetric matrix of n×n, where n is the number of well profiles for replicated retrieval, or the number of consensus profiles for MoA identification. Each element of the matrix is sampled

uniformly from $[-1, 1]$. Using true compound, or MoA, labels, we calculated the mean average precision and nearest-neighbor accuracy as described above. This procedure was repeated 5000 times, and we report the average metrics over all iterations.

2 Supplementary Tables

Model	Variant	Channels	Normalization	Accuracy	Macro F1	Micro F1
MAE-ProtS-CellS-Pool	DNA-Protein	DNA, Structure	Per Channel	*0.989 ± 0.0001*	0.871 ± 0.0027	0.989 ± 0.0001
			Per Image	*0.989 ± 0.0002*	0.867 ± 0.0026	0.989 ± 0.0002
	DNA-Protein-Concat	DNA, Structure, Plasma	Per Channel	**0.990 ± 0.0001**	**0.884 ± 0.0020**	**0.990 ± 0.0001**
			Per Image	*0.989 ± 0.0001*	0.861 ± 0.0020	*0.989 ± 0.0001*
	ER-DNA-Protein	DNA, Structure, Plasma	Per Channel	0.988 ± 0.0001	0.852 ± 0.0035	0.988 ± 0.0001
			Per Image	0.987 ± 0.0002	0.824 ± 0.0036	0.987 ± 0.0002
ViT-ProtS-Pool	DNA-Protein	DNA, Structure	Per Channel	0.981 ± 0.0007	0.685 ± 0.0306	0.981 ± 0.0007
			Per Image	0.980 ± 0.0006	0.662 ± 0.0245	0.980 ± 0.0006
	DNA-Protein-Concat	DNA, Structure, Plasma	Per Channel	0.985 ± 0.0010	0.755 ± 0.0291	0.985 ± 0.0010
			Per Image	0.984 ± 0.0010	0.740 ± 0.0306	0.984 ± 0.0010
	ER-DNA-Protein	DNA, Structure, Plasma	Per Channel	0.981 ± 0.0007	0.659 ± 0.0283	0.981 ± 0.0007
			Per Image	0.979 ± 0.0005	0.629 ± 0.0191	0.979 ± 0.0005
DINO4Cells-WTC-11	-	-	-	0.988 ± 0.0002	0.818 ± 0.0027	0.988 ± 0.0002
DINO4Cells-ImageNet	-	-	-	**0.990 ± 0.0002**	*0.882 ± 0.0036*	**0.990 ± 0.0002**

Table S1: Evaluation of SubCell Models with different channel combinations and normalizations on the AllenCell dataset.

Base Model	Prediction Head	Resize (pixels)	Cell-cycle			Localization		
			Acc	Macro F1	Macro AP	Acc	Macro F1	Macro AP
MAE-Cells-ProtS-Pool	LogReg	-	0.840	0.669	0.691	0.599	0.564	0.581
		128	0.889	0.734	0.786	0.659	0.627	0.665
		256	0.931	0.835	0.890	0.730	0.698	0.768
		512	0.946	**0.865**	0.903	0.747	0.721	0.789
	MLP	-	0.833	0.584	0.616	0.547	0.510	0.532
		128	0.887	0.719	0.759	0.677	0.645	0.697
		256	0.927	0.809	0.850	0.725	0.698	0.750
		512	0.929	0.825	0.860	0.729	0.700	0.760
ViT-ProtS-Pool	LogReg	-	0.840	0.596	0.700	0.588	0.548	0.578
		128	0.853	0.626	0.730	0.678	0.639	0.693
		256	0.852	0.595	0.691	0.685	0.649	0.698
		512	0.808	0.488	0.620	0.647	0.610	0.637
	MLP	-	0.844	0.667	0.703	0.571	0.527	0.562
		128	0.858	0.656	0.699	0.669	0.631	0.698
		256	0.872	0.662	0.709	0.689	0.652	0.703
		512	0.855	0.602	0.666	0.649	0.608	0.653
Pixel	LogReg	-	0.829	0.628	0.660	0.271	0.228	0.225
	MLP	-	0.874	0.746	0.784	0.466	0.414	0.432
CycleNet	-	-	**0.950**	**0.865**	**0.918**	-	-	-
DeepLoc	-	-	-	-	-	**0.788**	**0.757**	**0.825**

Table S2: Results for all models trained on the yeast datasets for cell-cycle stage prediction and protein localization predictions. Metrics shown are averaged over ten random seeds.

**Original Annotation**	**Low-level Annotations**	**High-level Annotations**	**OpenCell Annotations**
Actin filaments	Actin filaments	Cytoskeleton	cytoskeleton
Aggresome			
Cell Junctions	Plasma membrane	Plasma membrane	cell_contact
Centriolar satellite	Centrosome	Cytosol	centrosome
Centrosome	Centrosome	Cytoskeleton	centrosome
Cleavage furrow			
Cytokinetic bridge			
Cytoplasmic bodies	Cytosol	Cytosol	big_aggregates
Cytosol	Cytosol	Cytosol	cytoplasmic
Endoplasmic reticulum	Endoplasmic reticulum	Endomembrane system	er
Endosomes	Vesicles	Endomembrane system	vesicles
Focal adhesion sites	Actin filaments	Cytoskeleton	focal_adhesions
Golgi apparatus	Golgi apparatus	Endomembrane system	golgi
Intermediate filaments	Intermediate filaments	Cytoskeleton	cytoskeleton
Lipid droplets	Vesicles	Endomembrane system	vesicles
Lysosomes	Vesicles	Endomembrane system	vesicles
Microtubule ends	Microtubules	Cytoskeleton	cytoskeleton
Microtubules	Microtubules	Cytoskeleton	cytoskeleton
Midbody			
Midbody ring			
Mitochondria	Mitochondria	Mitochondria	mitochondria
Mitotic chromosome			
Mitotic spindle			
Nuclear bodies	Nucleus	Nucleus	nuclear_punctae
Nuclear membrane	Nuclear membrane	Nucleus	nuclear_membrane
Nuclear speckles	Nucleus	Nucleus	chromatin
Nucleoli	Nucleoli	Nucleoli	nucleolus_gc
Nucleoli fibrillar center	Nucleoli	Nucleoli	nucleolus_fc_dfc
Nucleoli rim	Nucleoli	Nucleoli	
Nucleoplasm	Nucleus	Nucleus	nucleoplasm
Peroxisomes	Vesicles	Endomembrane system	vesicles
Plasma membrane	Plasma membrane	Plasma membrane	membrane
Rods & Rings			
Vesicles	Vesicles	Endomembrane system	vesicles

Table S3: Original localization categories in the HPA dataset with their low-level, high-level, and OpenCell grouping annotated by experts.

Dataset	Subset	Proteins	FoVs	Cells	Pixel Size (μm)
HPAv23	Train	10,433	58,480	812,688	0.0801
	Validation	1,487	8,428	103,980	0.0801
	Test	3,460	18,041	221,358	0.0801
Kaggle	Test	851	1,112	21,880	0.0801
OpenCell	Test	1,310	6,301	94,426	0.2063
AllenCell	Train	25	18,079	136,983	0.1083
	Validation	25	15,260	34,246	0.1083
	Test	25	16,217	42,808	0.1083
Yeast(Cell Cycle)	Train	NA	NA	6,633	0.1077
	Test			764	0.1077
Yeast(Protein Localization)	Train	NA	NA	16,305	0.1077
	Test			1,824	0.1077
U2OS FUCCI	Test	1,166	2,798	357,083	0.1625
Bridge2AI	Test	474	1,404	131,206	0.0700
JUMP	Test	NA	3,456	1,734,123	0.3000

Table S4: Distribution of datasets into training, validation, and test sets used in this work.

A.

Dataset	Input Channels	Experiment	Macro AP	Micro AP	Label Rank. AP	Coverage Error
HPAv23	DNA-Protein	ViT-ProtS-Pool	0.673 ± 0.0033	0.856 ± 0.0008	0.888 ± 0.0005	2.077 ± 0.0046
	DNA-Protein	B8-ViT-ProtS-Pool	**0.732 ± 0.0022**	**0.875 ± 0.0011**	**0.901 ± 0.001**	**1.961 ± 0.0054**
	ER-DNA-Protein	ViT-ProtS-Pool	0.701 ± 0.0025	0.867 ± 0.0005	0.896 ± 0.0004	2.003 ± 0.0027
	ER-DNA-Protein	B8-ViT-ProtS-Pool	**0.745 ± 0.0041**	**0.88 ± 0.0011**	**0.906 ± 0.0009**	**1.926 ± 0.006**
	MT-DNA-Protein	ViT-ProtS-Pool	0.715 ± 0.0028	0.868 ± 0.0011	0.896 ± 0.0008	1.989 ± 0.0061
	MT-DNA-Protein	B8-ViT-ProtS-Pool	**0.762 ± 0.0042**	**0.882 ± 0.001**	**0.907 ± 0.0009**	**1.902 ± 0.0052**
	All Channels	ViT-ProtS-Pool	**0.76 ± 0.002**	0.881 ± 0.0008	**0.908 ± 0.0007**	**1.91 ± 0.0039**
	All Channels	B8-ViT-ProtS-Pool	0.755 ± 0.0038	0.881 ± 0.0008	0.906 ± 0.0006	1.914 ± 0.0041
Kaggle	DNA-Protein	ViT-ProtS-Pool	0.497 ± 0.003	0.61 ± 0.0009	0.699 ± 0.0007	4.201 ± 0.0128
	DNA-Protein	B8-ViT-ProtS-Pool	**0.535 ± 0.0024**	**0.625 ± 0.0022**	**0.709 ± 0.002**	**4.05 ± 0.0397**
	ER-DNA-Protein	ViT-ProtS-Pool	0.521 ± 0.0013	0.626 ± 0.0021	0.714 ± 0.0025	4.052 ± 0.0326
	ER-DNA-Protein	B8-ViT-ProtS-Pool	**0.548 ± 0.0025**	**0.634 ± 0.0022**	**0.718 ± 0.0022**	**3.978 ± 0.0323**
	MT-DNA-Protein	ViT-ProtS-Pool	0.534 ± 0.0034	0.654 ± 0.0007	0.732 ± 0.0011	3.811 ± 0.0169
	MT-DNA-Protein	B8-ViT-ProtS-Pool	**0.571 ± 0.0027**	**0.657 ± 0.0023**	**0.734 ± 0.0019**	**3.792 ± 0.043**
	All Channels	ViT-ProtS-Pool	0.565 ± 0.002	0.651 ± 0.0013	0.731 ± 0.0012	3.824 ± 0.0386
	All Channels	B8-ViT-ProtS-Pool	**0.563 ± 0.002**	**0.656 ± 0.0017**	**0.735 ± 0.002**	**3.774 ± 0.031**

B.

Dataset	Input Channels	Experiment	Macro AP	Micro AP
HPAv23	DNA-Protein	ViT-ProtS-Pool	0.646 ± 0.0085	0.87 ± 0.0033
	DNA-Protein	B8-ViT-ProtS-Pool	**0.856 ± 0.0028**	**0.959 ± 0.0004**
	ER-DNA-Protein	ViT-ProtS-Pool	0.865 ± 0.005	0.961 ± 0.001
	ER-DNA-Protein	B8-ViT-ProtS-Pool	**0.952 ± 0.0013**	**0.987 ± 0.0002**
	MT-DNA-Protein	ViT-ProtS-Pool	0.79 ± 0.0089	0.939 ± 0.0015
	MT-DNA-Protein	B8-ViT-ProtS-Pool	**0.928 ± 0.0014**	**0.982 ± 0.0002**
	All Channels	ViT-ProtS-Pool	**0.976 ± 0.0011**	**0.993 ± 0.0001**
	All Channels	B8-ViT-ProtS-Pool	0.952 ± 0.0015	0.987 ± 0.0002
Kaggle	DNA-Protein	ViT-ProtS-Pool	0.641 ± 0.0081	0.727 ± 0.0058
	DNA-Protein	B8-ViT-ProtS-Pool	**0.836 ± 0.0025**	**0.888 ± 0.0014**
	ER-DNA-Protein	ViT-ProtS-Pool	0.855 ± 0.0043	0.898 ± 0.003
	ER-DNA-Protein	B8-ViT-ProtS-Pool	**0.938 ± 0.0018**	**0.96 ± 0.0007**
	MT-DNA-Protein	ViT-ProtS-Pool	0.77 ± 0.0059	0.837 ± 0.0047
	MT-DNA-Protein	B8-ViT-ProtS-Pool	**0.904 ± 0.0022**	**0.938 ± 0.0006**
	All Channels	ViT-ProtS-Pool	**0.968 ± 0.0009**	**0.98 ± 0.0005**
	All Channels	B8-ViT-ProtS-Pool	0.939 ± 0.0026	0.958 ± 0.001

Table S5: Results of the models trained with a patch size of 8. Results for the ViT-ProtS-Pool model trained with the patch size of 8 on (A) localization prediction and (B) cell-line classification tasks.

A.

Dataset	Input Channels	Experiment	Macro AP	Micro AP	Label Rank. AP	Coverage Error
HPAv23	DNA-Protein	ViT-ProtS-Pool	0.673 ± 0.0033	0.856 ± 0.0008	0.888 ± 0.0005	2.077 ± 0.0046
	DNA-Protein	L16-ViT-ProtS-Pool	**0.736 ± 0.0024**	**0.877 ± 0.0009**	**0.903 ± 0.0007**	**1.948 ± 0.005**
	ER-DNA-Protein	ViT-ProtS-Pool	0.701 ± 0.0025	0.867 ± 0.0005	0.896 ± 0.0004	2.003 ± 0.0027
	ER-DNA-Protein	L16-ViT-ProtS-Pool	**0.741 ± 0.0033**	**0.88 ± 0.0015**	**0.906 ± 0.0013**	**1.93 ± 0.0064**
	MT-DNA-Protein	ViT-ProtS-Pool	0.715 ± 0.0028	0.868 ± 0.0011	0.896 ± 0.0008	1.989 ± 0.0061
	MT-DNA-Protein	L16-ViT-ProtS-Pool	**0.766 ± 0.0036**	**0.883 ± 0.0013**	**0.907 ± 0.0012**	**1.903 ± 0.0055**
	All Channels	ViT-ProtS-Pool	0.76 ± 0.002	0.881 ± 0.0008	**0.908 ± 0.0007**	1.91 ± 0.0039
	All Channels	L16-ViT-ProtS-Pool	**0.762 ± 0.0042**	**0.882 ± 0.0013**	0.907 ± 0.001	**1.905 ± 0.0052**
Kaggle	DNA-Protein	ViT-ProtS-Pool	0.497 ± 0.003	0.61 ± 0.0009	0.699 ± 0.0007	4.201 ± 0.0128
	DNA-Protein	L16-ViT-ProtS-Pool	**0.54 ± 0.0036**	**0.63 ± 0.0021**	**0.715 ± 0.0021**	**4.003 ± 0.0291**
	ER-DNA-Protein	ViT-ProtS-Pool	0.521 ± 0.0013	0.626 ± 0.0021	0.714 ± 0.0025	4.052 ± 0.0326
	ER-DNA-Protein	L16-ViT-ProtS-Pool	**0.544 ± 0.0028**	**0.636 ± 0.0016**	**0.72 ± 0.0014**	**3.954 ± 0.0222**
	MT-DNA-Protein	ViT-ProtS-Pool	0.534 ± 0.0034	0.654 ± 0.0007	0.732 ± 0.0011	3.811 ± 0.0169
	MT-DNA-Protein	L16-ViT-ProtS-Pool	**0.575 ± 0.0038**	**0.661 ± 0.0014**	**0.738 ± 0.0017**	**3.718 ± 0.0217**
	All Channels	ViT-ProtS-Pool	0.565 ± 0.002	0.651 ± 0.0013	0.731 ± 0.0012	3.824 ± 0.0386
	All Channels	L16-ViT-ProtS-Pool	**0.575 ± 0.0024**	**0.657 ± 0.0022**	**0.736 ± 0.0021**	**3.758 ± 0.0398**

B.

Dataset	Input Channels	Experiment	Macro AP	Micro AP
HPAv23	DNA-Protein	ViT-ProtS-Pool	0.646 ± 0.0085	0.87 ± 0.0033
	DNA-Protein	L16-ViT-ProtS-Pool	**0.838 ± 0.0038**	**0.956 ± 0.0005**
	ER-DNA-Protein	ViT-ProtS-Pool	0.865 ± 0.005	0.961 ± 0.001
	ER-DNA-Protein	L16-ViT-ProtS-Pool	**0.933 ± 0.0028**	**0.984 ± 0.0004**
	MT-DNA-Protein	ViT-ProtS-Pool	0.79 ± 0.0089	0.939 ± 0.0015
	MT-DNA-Protein	L16-ViT-ProtS-Pool	**0.91 ± 0.0029**	**0.979 ± 0.0003**
	All Channels	ViT-ProtS-Pool	**0.976 ± 0.0011**	**0.993 ± 0.0001**
	All Channels	L16-ViT-ProtS-Pool	0.962 ± 0.0011	0.991 ± 0.0001
Kaggle	DNA-Protein	ViT-ProtS-Pool	0.641 ± 0.0081	0.727 ± 0.0058
	DNA-Protein	L16-ViT-ProtS-Pool	**0.829 ± 0.0016**	**0.875 ± 0.0012**
	ER-DNA-Protein	ViT-ProtS-Pool	0.855 ± 0.0043	0.898 ± 0.003
	ER-DNA-Protein	L16-ViT-ProtS-Pool	**0.923 ± 0.0031**	**0.95 ± 0.001**
	MT-DNA-Protein	ViT-ProtS-Pool	0.77 ± 0.0059	0.837 ± 0.0047
	MT-DNA-Protein	L16-ViT-ProtS-Pool	**0.889 ± 0.0036**	**0.925 ± 0.0016**
	All Channels	ViT-ProtS-Pool	**0.968 ± 0.0009**	**0.98 ± 0.0005**
	All Channels	L16-ViT-ProtS-Pool	0.954 ± 0.0014	0.969 ± 0.0006

Table S6: Results of the ViT-Large model. Results for the ViT-L model ViT-ProtS-Pool configuration on (A) localization prediction and (B) cell-line classification tasks.

A.

Dataset	Experiment	Macro AP	Micro AP	Label Rank. AP	Coverage Error
HPAv23	MAE-MR0.25-CMR-0.0	0.518 ± 0.0039	0.775 ± 0.0014	0.827 ± 0.001	2.57 ± 0.0084
	MAE-MR0.25-CMR-0.25	0.514 ± 0.0037	0.773 ± 0.0013	0.825 ± 0.0009	2.58 ± 0.0091
	MAE-MR0.25-CMR-0.5	*0.523 ± 0.0035*	*0.778 ± 0.0013*	*0.829 ± 0.0013*	*2.551 ± 0.0124*
	MAE-MR0.33-CMR-0.0	0.515 ± 0.0043	0.772 ± 0.0018	0.824 ± 0.0013	2.591 ± 0.0121
	MAE-MR0.33-CMR-0.25	0.519 ± 0.0027	0.775 ± 0.001	0.827 ± 0.001	2.568 ± 0.0096
	MAE-MR0.33-CMR-0.5	**0.528 ± 0.0045**	**0.78 ± 0.0017**	**0.831 ± 0.0013**	**2.535 ± 0.0114**
	MAE-MR0.5-CMR-0.0	0.493 ± 0.0039	0.76 ± 0.0017	0.816 ± 0.0015	2.663 ± 0.011
	MAE-MR0.5-CMR-0.25	0.493 ± 0.0036	0.765 ± 0.0011	0.818 ± 0.0011	2.635 ± 0.0082
	MAE-MR0.5-CMR-0.5	0.512 ± 0.0036	0.773 ± 0.0013	0.826 ± 0.0009	2.573 ± 0.0092
	DINO4Cells-HPA	**0.705 ± 0.0022**	**0.863 ± 0.001**	**0.895 ± 0.0007**	**2.013 ± 0.0059**
Kaggle	MAE-MR0.25-CMR-0.0	0.355 ± 0.0036	0.545 ± 0.0013	0.652 ± 0.0011	4.895 ± 0.0161
	MAE-MR0.25-CMR-0.25	0.348 ± 0.0031	0.544 ± 0.0017	0.65 ± 0.0016	4.947 ± 0.0227
	MAE-MR0.25-CMR-0.5	*0.361 ± 0.0019*	**0.552 ± 0.0011**	**0.657 ± 0.0016**	**4.838 ± 0.0237**
	MAE-MR0.33-CMR-0.0	0.354 ± 0.0033	0.544 ± 0.0019	0.651 ± 0.0016	4.919 ± 0.024
	MAE-MR0.33-CMR-0.25	0.355 ± 0.0032	0.547 ± 0.0014	0.652 ± 0.0017	*4.872 ± 0.0263*
	MAE-MR0.33-CMR-0.5	**0.363 ± 0.0035**	*0.548 ± 0.0018*	*0.654 ± 0.0015*	4.873 ± 0.0244
	MAE-MR0.5-CMR-0.0	0.329 ± 0.0035	0.53 ± 0.0023	0.639 ± 0.002	5.012 ± 0.0263
	MAE-MR0.5-CMR-0.25	0.335 ± 0.0027	0.529 ± 0.0018	0.638 ± 0.0017	5.03 ± 0.0284
	MAE-MR0.5-CMR-0.5	0.349 ± 0.003	0.54 ± 0.0022	0.645 ± 0.0022	4.941 ± 0.0276
	DINO4Cells-HPA	**0.519 ± 0.004**	**0.631 ± 0.0033**	**0.718 ± 0.0026**	**4.047 ± 0.0381**

B.

Dataset	Experiment	Macro AP	Micro AP
HPAv23	MAE-MR0.25-CMR-0.0	0.923 ± 0.0073	0.977 ± 0.0014
	MAE-MR0.25-CMR-0.25	0.93 ± 0.0031	0.978 ± 0.0007
	MAE-MR0.25-CMR-0.5	0.935 ± 0.0042	0.981 ± 0.0008
	MAE-MR0.33-CMR-0.0	0.924 ± 0.003	0.979 ± 0.0006
	MAE-MR0.33-CMR-0.25	0.928 ± 0.0042	0.979 ± 0.0009
	MAE-MR0.33-CMR-0.5	*0.937 ± 0.0047*	*0.981 ± 0.0007*
	MAE-MR0.5-CMR-0.0	**0.94 ± 0.0041**	**0.982 ± 0.0008**
	MAE-MR0.5-CMR-0.25	0.894 ± 0.0068	0.967 ± 0.0015
	MAE-MR0.5-CMR-0.5	0.926 ± 0.0034	0.978 ± 0.0006
	DINO4Cells-HPA	**0.957 ± 0.0014**	**0.99 ± 0.0002**
Kaggle	MAE-MR0.25-CMR-0.0	0.888 ± 0.0086	0.928 ± 0.0038
	MAE-MR0.25-CMR-0.25	0.905 ± 0.0037	0.937 ± 0.002
	MAE-MR0.25-CMR-0.5	0.907 ± 0.0045	0.941 ± 0.003
	MAE-MR0.33-CMR-0.0	0.895 ± 0.0035	0.932 ± 0.002
	MAE-MR0.33-CMR-0.25	0.898 ± 0.0055	0.934 ± 0.0035
	MAE-MR0.33-CMR-0.5	*0.908 ± 0.0041*	*0.941 ± 0.003*
	MAE-MR0.5-CMR-0.0	**0.912 ± 0.0043**	**0.943 ± 0.0026**
	MAE-MR0.5-CMR-0.25	0.842 ± 0.0083	0.896 ± 0.0051
	MAE-MR0.5-CMR-0.5	0.892 ± 0.0027	0.932 ± 0.0022
	DINO4Cells-HPA	**0.95 ± 0.0014**	**0.964 ± 0.0014**

Table S7: Results for the MAE experiments for A.) localization prediction and B.) cell-line classification tasks. (MR refers to the overall masking ratio for the input image, and CMR refers to the masking ratio of the cells.)

A.

Dataset	Experiment	Macro AP	Micro AP	Label Rank. AP	Coverage Error
HPAv23	MAE-MR0.25-CMR-0.0-CellS	*0.656 ± 0.0021*	*0.845 ± 0.0006*	*0.881 ± 0.0004*	*2.112 ± 0.0032*
	MAE-MR0.25-CMR-0.5-CellS	0.648 ± 0.0025	0.842 ± 0.0004	0.879 ± 0.0005	2.133 ± 0.0027
	MAE-MR0.33-CMR-0.0-CellS	0.648 ± 0.0036	0.842 ± 0.0014	0.879 ± 0.0008	2.129 ± 0.0058
	MAE-MR0.33-CMR-0.5-CellS	0.641 ± 0.0012	0.84 ± 0.0004	0.878 ± 0.0006	2.136 ± 0.0041
	ViT-CellS	**0.666 ± 0.003**	**0.848 ± 0.0006**	**0.884 ± 0.0003**	**2.1 ± 0.0036**
	DINO4Cells-HPA	**0.705 ± 0.0022**	**0.863 ± 0.001**	**0.895 ± 0.0007**	**2.013 ± 0.0059**
Kaggle	MAE-MR0.25-CMR-0.0-CellS	*0.502 ± 0.0032*	*0.623 ± 0.0014*	*0.715 ± 0.0012*	*4.023 ± 0.0171*
	MAE-MR0.25-CMR-0.5-CellS	0.498 ± 0.0015	0.621 ± 0.0012	*0.715 ± 0.0014*	4.016 ± 0.0163
	MAE-MR0.33-CMR-0.0-CellS	0.498 ± 0.0029	0.621 ± 0.0013	*0.715 ± 0.0009*	4.031 ± 0.0214
	MAE-MR0.33-CMR-0.5-CellS	0.488 ± 0.004	0.616 ± 0.0015	0.713 ± 0.0013	4.06 ± 0.0113
	ViT-CellS	**0.509 ± 0.0032**	**0.625 ± 0.0012**	**0.717 ± 0.0013**	**3.975 ± 0.0166**
	DINO4Cells-HPA	**0.519 ± 0.004**	**0.631 ± 0.0033**	**0.718 ± 0.0026**	**4.047 ± 0.0381**

B.

Dataset	Experiment	Macro AP	Micro AP
HPAv23	MAE-MR0.25-CMR-0.0-CellS	0.939 ± 0.0014	0.985 ± 0.0002
	MAE-MR0.25-CMR-0.5-CellS	**0.941 ± 0.0017**	**0.987 ± 0.0003**
	MAE-MR0.33-CMR-0.0-CellS	0.94 ± 0.0022	*0.986 ± 0.0003*
	MAE-MR0.33-CMR-0.5-CellS	**0.941 ± 0.0014**	*0.986 ± 0.0003*
	ViT-CellS	0.916 ± 0.0036	0.98 ± 0.0005
	DINO4Cells-HPA	**0.957 ± 0.0014**	**0.99 ± 0.0002**
Kaggle	MAE-MR0.25-CMR-0.0-CellS	0.919 ± 0.0016	0.944 ± 0.0007
	MAE-MR0.25-CMR-0.5-CellS	**0.929 ± 0.0015**	**0.948 ± 0.0005**
	MAE-MR0.33-CMR-0.0-CellS	0.923 ± 0.0017	*0.946 ± 0.0008*
	MAE-MR0.33-CMR-0.5-CellS	*0.924 ± 0.0014*	*0.946 ± 0.0009*
	ViT-CellS	0.894 ± 0.005	0.923 ± 0.0022
	DINO4Cells-HPA	**0.95 ± 0.0014**	**0.964 ± 0.0014**

Table S8: Results for the cell-specific experiments for (A) localization prediction and (B) cell-line classification tasks.

A.

Dataset	Experiment	Macro AP	Micro AP	Label Rank. AP	Coverage Error
HPAv23	ViT-ProtS	**0.742 ± 0.0047**	**0.878 ± 0.0013**	**0.903 ± 0.0012**	**1.934 ± 0.008**
	ViT-CellS-ProtS	*0.728 ± 0.0031*	*0.872 ± 0.0004*	0.899 ± 0.0004	1.969 ± 0.0027
	MAE-MR0.25-CMR-0.0-ProtS	0.709 ± 0.0046	0.858 ± 0.0012	0.893 ± 0.0004	2.03 ± 0.0049
	MAE-MR0.25-CMR-0.5-ProtS	0.707 ± 0.002	0.856 ± 0.0008	0.891 ± 0.0006	2.034 ± 0.0044
	MAE-MR0.25-CMR-0.0-CellS-ProtS	*0.729 ± 0.0037*	*0.872 ± 0.0011*	*0.901 ± 0.0012*	*1.959 ± 0.0055*
	MAE-MR0.25-CMR-0.5-CellS-ProtS	0.719 ± 0.0036	0.869 ± 0.0006	0.899 ± 0.0007	1.974 ± 0.0049
	DINO4Cells-HPA	0.705 ± 0.0022	0.863 ± 0.001	0.895 ± 0.0007	2.013 ± 0.0059
	ViT-Weak-Supervised	0.734 ± 0.0008	0.869 ± 0.0004	0.898 ± 0.0004	1.985 ± 0.0029
	bestfitting	**0.77**	**0.83**	**0.877**	**2.029**
Kaggle	ViT-ProtS	**0.559 ± 0.0058**	**0.653 ± 0.0017**	*0.733 ± 0.0026*	*3.823 ± 0.0464*
	ViT-CellS-ProtS	*0.552 ± 0.0035*	*0.652 ± 0.0015*	**0.734 ± 0.0012**	**3.813 ± 0.0186**
	MAE-MR0.25-CMR-0.0-ProtS	0.506 ± 0.0054	0.614 ± 0.0067	0.708 ± 0.0044	4.031 ± 0.0478
	MAE-MR0.25-CMR-0.5-ProtS	0.505 ± 0.0043	0.61 ± 0.0034	0.705 ± 0.0028	4.035 ± 0.0404
	MAE-MR0.25-CMR-0.0-CellS-ProtS	0.542 ± 0.0036	0.64 ± 0.0032	0.725 ± 0.0024	3.903 ± 0.0352
	MAE-MR0.25-CMR-0.5-CellS-ProtS	0.53 ± 0.0032	0.631 ± 0.0026	0.718 ± 0.0019	4.012 ± 0.0264
	DINO4Cells-HPA	0.519 ± 0.004	0.631 ± 0.0033	0.718 ± 0.0026	4.047 ± 0.0381
	ViT-Weak-Supervised	0.544 ± 0.0005	0.644 ± 0.001	0.727 ± 0.001	3.914 ± 0.0172
	bestfitting	**0.606**	**0.663**	**0.768**	**3.049**

B.

Dataset	Experiment	Macro AP	Micro AP
HPAv23	ViT-ProtS	0.959 ± 0.0025	0.99 ± 0.0003
	ViT-CellS-ProtS	0.958 ± 0.0019	0.99 ± 0.0002
	MAE-MR0.25-CMR-0.0-ProtS	0.956 ± 0.0008	0.986 ± 0.0001
	MAE-MR0.25-CMR-0.5-ProtS	0.958 ± 0.0007	0.988 ± 0.0001
	MAE-MR0.25-CMR-0.0-CellS-ProtS	**0.974 ± 0.0009**	**0.994 ± 0.0001**
	MAE-MR0.25-CMR-0.5-CellS-ProtS	*0.972 ± 0.0006*	*0.993 ± 0.0*
	DINO4Cells-HPA	**0.957 ± 0.0014**	**0.99 ± 0.0002**
	ViT-Weak-Supervised	0.552 ± 0.0103	0.813 ± 0.0034
	bestfitting	0.342 ± 0.0038	0.682 ± 0.0013
Kaggle	ViT-ProtS	0.948 ± 0.0024	0.968 ± 0.0009
	ViT-CellS-ProtS	0.949 ± 0.0019	0.964 ± 0.001
	MAE-MR0.25-CMR-0.0-ProtS	0.93 ± 0.001	0.953 ± 0.0009
	MAE-MR0.25-CMR-0.5-ProtS	0.94 ± 0.001	0.957 ± 0.0006
	MAE-MR0.25-CMR-0.0-CellS-ProtS	**0.961 ± 0.0009**	**0.976 ± 0.0005**
	MAE-MR0.25-CMR-0.5-CellS-ProtS	*0.957 ± 0.0011*	0.969 ± 0.0004
	DINO4Cells-HPA	**0.95 ± 0.0014**	**0.964 ± 0.0014**
	ViT-Weak-Supervised	0.531 ± 0.0058	0.613 ± 0.0046
	bestfitting	0.316 ± 0.0037	0.42 ± 0.0029

Table S9: Results for the protein-specific experiments for (A) localization classification and (B) cell-line classification tasks.

A.

Dataset	Experiment	Macro AP	Micro AP	Label Rank. AP	Coverage Error
HPAv23	ViT-ProtS	*0.742 ± 0.0047*	*0.878 ± 0.0013*	0.903 ± 0.0012	1.934 ± 0.008
	ViT-ProtS-Pool	**0.76 ± 0.002**	**0.881 ± 0.0008**	**0.908 ± 0.0007**	**1.91 ± 0.0039**
	MAE-MR0.25-CMR-0.0-CellS-ProtS	0.729 ± 0.0037	0.872 ± 0.0011	0.901 ± 0.0012	1.959 ± 0.0055
	MAE-MR0.25-CMR-0.0-CellS-ProtS-Pool	0.739 ± 0.0024	0.876 ± 0.0004	*0.904 ± 0.0005*	*1.941 ± 0.0038*
	DINO4Cells-HPA	0.705 ± 0.0022	0.863 ± 0.001	0.895 ± 0.0007	2.013 ± 0.0059
	ViT-Weak-Supervised	0.734 ± 0.0008	**0.869 ± 0.0004**	**0.898 ± 0.0004**	**1.985 ± 0.0029**
	bestfitting	**0.77**	0.83	0.877	2.029
Kaggle	ViT-ProtS	0.559 ± 0.0058	**0.653 ± 0.0017**	**0.733 ± 0.0026**	**3.823 ± 0.0464**
	ViT-ProtS-Pool	**0.565 ± 0.002**	*0.651 ± 0.0013*	*0.731 ± 0.0012*	*3.824 ± 0.0386*
	MAE-MR0.25-CMR-0.0-CellS-ProtS	0.542 ± 0.0036	0.64 ± 0.0032	0.725 ± 0.0024	3.903 ± 0.0352
	MAE-MR0.25-CMR-0.0-CellS-ProtS-Pool	0.552 ± 0.0018	0.647 ± 0.0027	0.73 ± 0.0024	*3.87 ± 0.0379*
	DINO4Cells-HPA	0.519 ± 0.004	0.631 ± 0.0033	0.718 ± 0.0026	4.047 ± 0.0381
	ViT-Weak-Supervised	0.544 ± 0.0005	0.644 ± 0.001	0.727 ± 0.001	3.914 ± 0.0172
	bestfitting	**0.606**	**0.663**	**0.768**	**3.049**

B.

Dataset	Experiment	Macro AP	Micro AP
HPAv23	ViT-ProtS	0.959 ± 0.0025	0.99 ± 0.0003
	ViT-ProtS-Pool	**0.976 ± 0.0011**	0.993 ± 0.0001
	MAE-MR0.25-CMR-0.0-CellS-ProtS	0.974 ± 0.0009	*0.994 ± 0.0001*
	MAE-MR0.25-CMR-0.0-CellS-ProtS-Pool	**0.976 ± 0.0009**	**0.995 ± 0.0001**
	DINO4Cells-HPA	**0.957 ± 0.0014**	**0.99 ± 0.0002**
	ViT-Weak-Supervised	0.552 ± 0.0103	0.813 ± 0.0034
	bestfitting	0.342 ± 0.0038	0.682 ± 0.0013
Kaggle	ViT-ProtS	0.948 ± 0.0024	0.968 ± 0.0009
	ViT-ProtS-Pool	**0.968 ± 0.0009**	**0.98 ± 0.0005**
	MAE-MR0.25-CMR-0.0-CellS-ProtS	0.961 ± 0.0009	0.976 ± 0.0005
	MAE-MR0.25-CMR-0.0-CellS-ProtS-Pool	*0.965 ± 0.0013*	*0.977 ± 0.0006*
	DINO4Cells-HPA	**0.95 ± 0.0014**	**0.964 ± 0.0014**
	ViT-Weak-Supervised	0.531 ± 0.0058	0.613 ± 0.0046
	bestfitting	0.316 ± 0.0037	0.42 ± 0.0029

Table S10: Results for evaluating the impact of the attention pooling module on the best models for (A) localization prediction and (B) cell-line classification tasks.

Dataset	Experiment	Macro AP	Micro AP	Label Rank. AP	Coverage Error
HPAv23	ViT-ProtS	*0.557 ± 0.0026*	*0.857 ± 0.0004*	*0.889 ± 0.0006*	*2.214 ± 0.004*
	ViT-ProtS-Pool	**0.577 ± 0.0018**	**0.861 ± 0.0006**	**0.893 ± 0.0008**	**2.18 ± 0.0058**
	MAE-MR0.25-CMR-0.0-CellS-ProtS	0.547 ± 0.0022	0.851 ± 0.0005	0.887 ± 0.0008	2.244 ± 0.0048
	MAE-MR0.25-CMR-0.0-CellS-ProtS-Pool	0.553 ± 0.0022	0.856 ± 0.0005	*0.889 ± 0.0006*	2.225 ± 0.0052
	DINO4Cells-HPA	0.509 ± 0.0033	0.839 ± 0.0009	0.878 ± 0.0007	2.36 ± 0.0078
	ViT-Weak-Supervised	0.534 ± 0.0013	0.847 ± 0.0004	0.882 ± 0.0004	2.316 ± 0.0028
	bestfitting	**0.569 ± 0.0022**	**0.856 ± 0.0007**	**0.888 ± 0.0004**	**2.228 ± 0.0029**
Kaggle	ViT-ProtS	*0.42 ± 0.0017*	**0.636 ± 0.0009**	**0.719 ± 0.0013**	**4.45 ± 0.0323**
	ViT-ProtS-Pool	**0.427 ± 0.003**	*0.634 ± 0.0014*	*0.716 ± 0.0015*	*4.466 ± 0.0522*
	MAE-MR0.25-CMR-0.0-CellS-ProtS	0.398 ± 0.0025	0.623 ± 0.0022	0.711 ± 0.0021	4.557 ± 0.0423
	MAE-MR0.25-CMR-0.0-CellS-ProtS-Pool	0.41 ± 0.0021	0.631 ± 0.0014	0.715 ± 0.0013	4.527 ± 0.0332
	DINO4Cells-HPA	0.376 ± 0.0026	0.611 ± 0.0047	0.7 ± 0.0035	4.863 ± 0.0761
	ViT-Weak-Supervised	0.399 ± 0.0016	0.627 ± 0.0009	0.712 ± 0.0007	4.565 ± 0.0211
	bestfitting	**0.428 ± 0.0021**	**0.642 ± 0.0019**	**0.725 ± 0.0015**	**4.364 ± 0.0278**

Table S11: Evaluating the performance of the models on a broader range of localization categories. Classification results of the models when evaluating the features on the 31 categories present in the HPA dataset.

A.

Dataset	Experiment	Macro AP	Micro AP	Label Rank. AP	Coverage Error
HPAv23	ViT-BYOL	0.719 ± 0.0011	0.868 ± 0.0013	0.898 ± 0.0013	1.997 ± 0.0092
	ViT-Contrast	**0.742 ± 0.0047**	**0.878 ± 0.0013**	**0.903 ± 0.0012**	**1.934 ± 0.008**
	ViT-CellS-BYOL	**0.732 ± 0.0024**	0.872 ± 0.0013	**0.901 ± 0.0011**	**1.959 ± 0.0063**
	ViT-CellS-Contrast	0.728 ± 0.0031	0.872 ± 0.0004	0.899 ± 0.0004	1.969 ± 0.0027
	MAE-MR0.25-CMR-0.0-BYOL	0.684 ± 0.0019	0.852 ± 0.0007	0.888 ± 0.0007	2.071 ± 0.0108
	MAE-MR0.25-CMR-0.0-Contrast	**0.709 ± 0.0046**	**0.858 ± 0.0012**	**0.893 ± 0.0004**	**2.03 ± 0.0049**
	MAE-MR0.25-CMR-0.5-BYOL	0.685 ± 0.0023	0.852 ± 0.001	0.887 ± 0.0007	2.076 ± 0.0048
	MAE-MR0.25-CMR-0.5-Contrast	**0.707 ± 0.002**	**0.856 ± 0.0008**	**0.891 ± 0.0006**	**2.034 ± 0.0044**
	MAE-MR0.25-CMR-0.0-CellS-BYOL	0.696 ± 0.005	0.862 ± 0.0015	0.893 ± 0.0015	2.032 ± 0.01
	MAE-MR0.25-CMR-0.0-CellS-Contrast	**0.729 ± 0.0037**	**0.872 ± 0.0011**	**0.901 ± 0.0012**	**1.959 ± 0.0055**
	MAE-MR0.25-CMR-0.5-CellS-BYOL	0.71 ± 0.002	0.862 ± 0.001	0.894 ± 0.0009	2.025 ± 0.0046
	MAE-MR0.25-CMR-0.5-CellS-Contrast	**0.719 ± 0.0036**	**0.869 ± 0.0006**	**0.899 ± 0.0007**	**1.974 ± 0.0049**
	bestfitting	**0.77**	**0.83**	**0.877**	**2.029**
Kaggle	ViT-BYOL	0.523 ± 0.0018	0.63 ± 0.0012	0.717 ± 0.001	4.022 ± 0.0316
	ViT-Contrast	**0.559 ± 0.0058**	**0.653 ± 0.0017**	**0.733 ± 0.0026**	**3.823 ± 0.0464**
	ViT-CellS-BYOL	0.536 ± 0.0032	0.641 ± 0.0018	0.724 ± 0.002	3.871 ± 0.0398
	ViT-CellS-Contrast	**0.552 ± 0.0035**	**0.652 ± 0.0015**	**0.734 ± 0.0012**	**3.813 ± 0.0186**
	MAE-MR0.25-CMR-0.0-BYOL	0.479 ± 0.0021	0.601 ± 0.0048	0.698 ± 0.0037	4.229 ± 0.05
	MAE-MR0.25-CMR-0.0-Contrast	**0.506 ± 0.0054**	**0.614 ± 0.0067**	**0.708 ± 0.0044**	**4.031 ± 0.0478**
	MAE-MR0.25-CMR-0.5-BYOL	0.469 ± 0.0031	0.599 ± 0.0028	0.698 ± 0.0019	4.244 ± 0.0228
	MAE-MR0.25-CMR-0.5-Contrast	**0.505 ± 0.0043**	**0.61 ± 0.0034**	**0.705 ± 0.0028**	**4.035 ± 0.0404**
	MAE-MR0.25-CMR-0.0-CellS-BYOL	0.501 ± 0.0031	0.622 ± 0.0017	0.712 ± 0.0016	4.054 ± 0.0188
	MAE-MR0.25-CMR-0.0-CellS-Contrast	**0.542 ± 0.0036**	**0.64 ± 0.0032**	**0.725 ± 0.0024**	**3.903 ± 0.0352**
	MAE-MR0.25-CMR-0.5-CellS-BYOL	0.5 ± 0.0041	0.612 ± 0.0022	0.706 ± 0.0014	4.068 ± 0.0291
	MAE-MR0.25-CMR-0.5-CellS-Contrast	**0.53 ± 0.0032**	**0.631 ± 0.0026**	**0.718 ± 0.0019**	**4.012 ± 0.0264**
	bestfitting	**0.606**	**0.663**	**0.768**	**3.049**

B.

Dataset	Experiment	Macro AP	Micro AP
HPAv23	ViT-BYOL	0.933 ± 0.0021	0.98 ± 0.0003
	ViT-Contrast	**0.959 ± 0.0025**	**0.99 ± 0.0003**
	ViT-CellS-BYOL	0.954 ± 0.0011	0.989 ± 0.0002
	ViT-CellS-Contrast	**0.958 ± 0.0019**	**0.99 ± 0.0002**
	MAE-MR0.25-CMR-0.0-BYOL	0.916 ± 0.0016	0.974 ± 0.0004
	MAE-MR0.25-CMR-0.0-Contrast	**0.956 ± 0.0008**	**0.986 ± 0.0001**
	MAE-MR0.25-CMR-0.5-BYOL	0.93 ± 0.0013	0.979 ± 0.0002
	MAE-MR0.25-CMR-0.5-Contrast	**0.958 ± 0.0007**	**0.988 ± 0.0001**
	MAE-MR0.25-CMR-0.0-CellS-BYOL	0.947 ± 0.0014	0.988 ± 0.0002
	MAE-MR0.25-CMR-0.0-CellS-Contrast	**0.974 ± 0.0009**	**0.994 ± 0.0001**
	MAE-MR0.25-CMR-0.5-CellS-BYOL	0.954 ± 0.001	0.989 ± 0.0001
	MAE-MR0.25-CMR-0.5-CellS-Contrast	**0.972 ± 0.0006**	**0.993 ± 0.0**
	bestfitting	0.342 ± 0.0038	0.682 ± 0.0013
Kaggle	ViT-BYOL	0.905 ± 0.0014	0.931 ± 0.001
	ViT-Contrast	**0.948 ± 0.0024**	**0.968 ± 0.0009**
	ViT-CellS-BYOL	0.939 ± 0.0011	0.957 ± 0.0008
	ViT-CellS-Contrast	**0.949 ± 0.0019**	**0.964 ± 0.001**
	MAE-MR0.25-CMR-0.0-BYOL	0.877 ± 0.0034	0.908 ± 0.0024
	MAE-MR0.25-CMR-0.0-Contrast	**0.93 ± 0.001**	**0.953 ± 0.0009**
	MAE-MR0.25-CMR-0.5-BYOL	0.893 ± 0.0015	0.925 ± 0.0018
	MAE-MR0.25-CMR-0.5-Contrast	**0.94 ± 0.001**	**0.957 ± 0.0006**
	MAE-MR0.25-CMR-0.0-CellS-BYOL	0.921 ± 0.0013	0.944 ± 0.0012
	MAE-MR0.25-CMR-0.0-CellS-Contrast	**0.961 ± 0.0009**	**0.976 ± 0.0005**
	MAE-MR0.25-CMR-0.5-CellS-BYOL	0.923 ± 0.0016	0.949 ± 0.0006
	MAE-MR0.25-CMR-0.5-CellS-Contrast	**0.957 ± 0.0011**	**0.969 ± 0.0004**
	bestfitting	0.316 ± 0.0037	0.42 ± 0.0029

Table S12: Evaluating BYOL as the protein-specific loss. Results for the different multitask configurations trained with the BYOL loss as the protein-specific loss on (A) localization prediction and (B) cell-line classification tasks.

3 Supplementary Figures

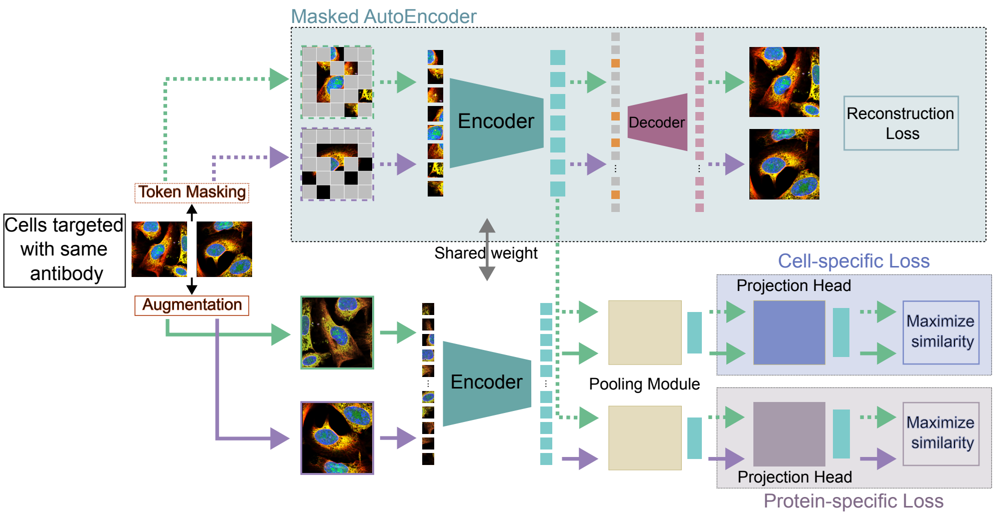

Fig. S1: A detailed overview of the multi-task learning framework (MAE-CellS-ProtS-Pool) used in the paper. A batch consisting of multiple images from different antibodies is constructed. One of the views is augmented with geometric augmentations and token masking and fed into the MAE branch. The other view is augmented with geometric and color augmentations and passed through a cell- and protein-specific branch. Finally, the cell-specific and protein-specific loss is calculated over the embeddings from both branches.

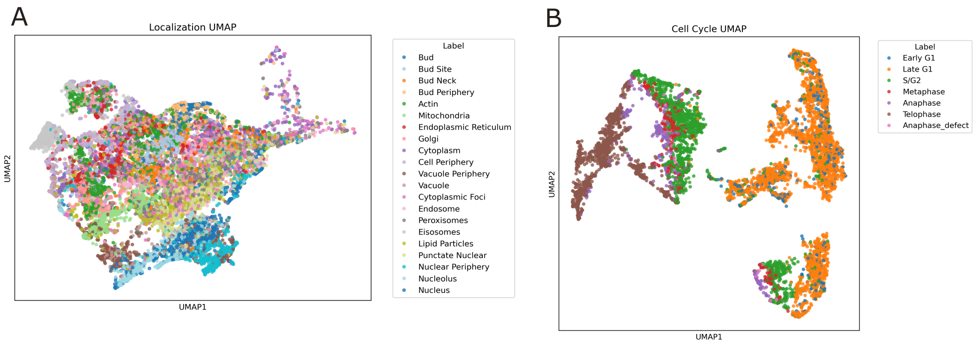

Fig. S2: UMAP of MAE-CellS-ProtS-Pool embeddings for yeast images resized to 512x512 (A) colored by protein localization and (B) colored by cell-cycle stage

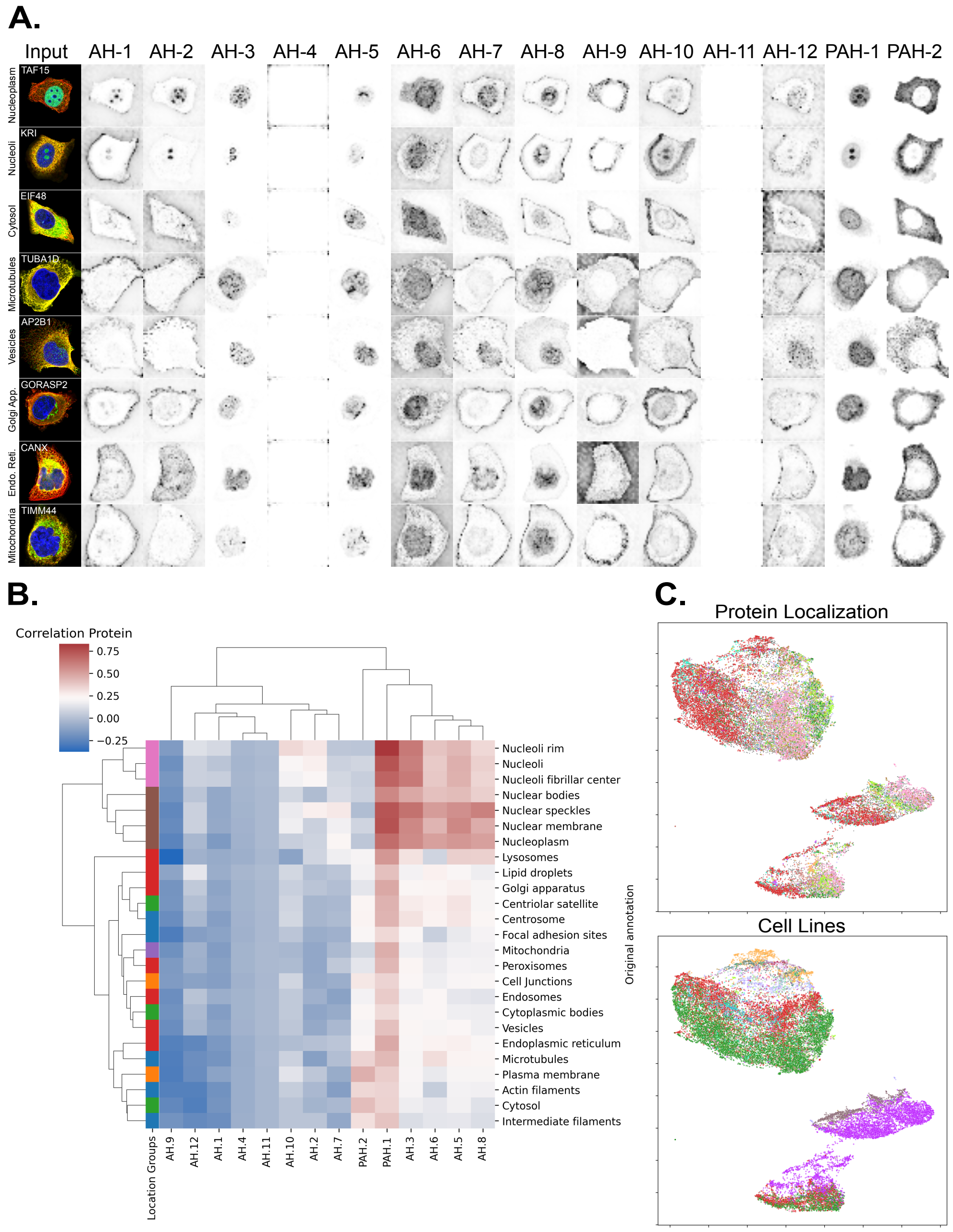

Fig. S3: A.) Examples of fluorescent images and the attention captured by the attention heads and pooled attention heads in the ViT-ProtS-Pool model for different protein localizations. The twelve attention heads of the vision transformers are marked by the prefix AH-, and the pooled attention heads by the prefix PAH-. B.) A cluster map showing the aggregated correlation profiles of the attention heads with the protein channel for different localization categories. The order of rows and columns is set by the hierarchical clustering of the correlation profiles. The positions of major location groups are shown in different colors on the left side of the plot. C.) The UMAP visualization of the correlation profiles for all fluorescent channels, along with the attention maps. The profiles were aggregated over the FOVs, and the colors represent protein localization groups (top) and cell lines (bottom).

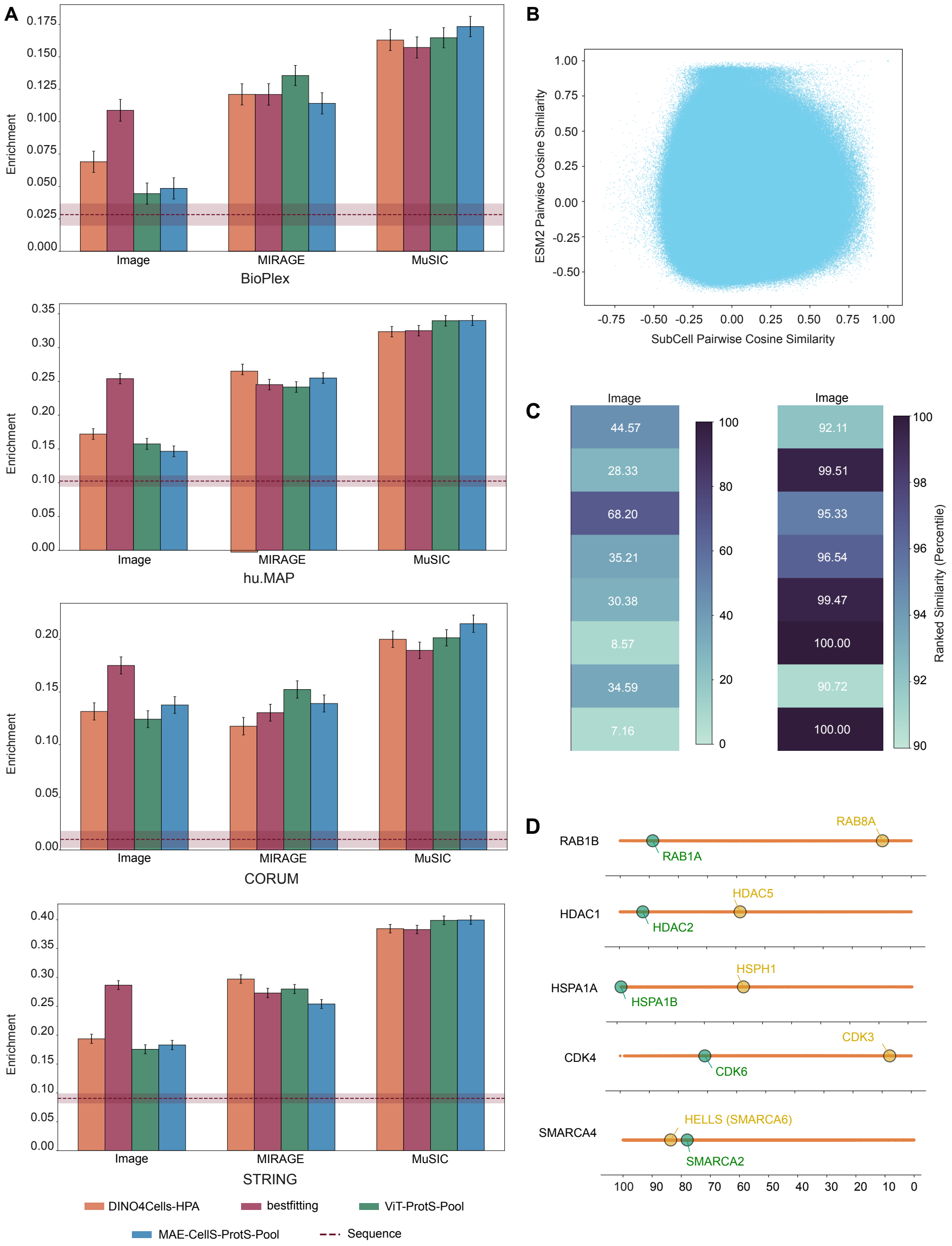

Fig. S4: Additional multimodal integration performance. A) Performance of different image models across interaction tasks, using different integration methods (image alone, co-embedded using the MIRAGE method, and co-embedding using the MuSIC method). B) The pairwise cosine similarity of SubCell MAE-CellS-ProtS-Pool vs. ESM2 embedding pairs for a sample of protein pairs. This data has an R^2 of –2.0. C) Heatmap tables displaying the similarity of embeddings for genes that are functionally divergent paralogs (left) and functionally redundant paralogs (right) in the SubCell MAE-CellS-ProtS-Pool space. Here, similarity is quantified the same way as in Figure 6D. D) Number lines displaying comparative differences in the similarity between functionally divergent paralogs (yellow) and functionally redundant paralogs (green) of a gene common between the two in image space.

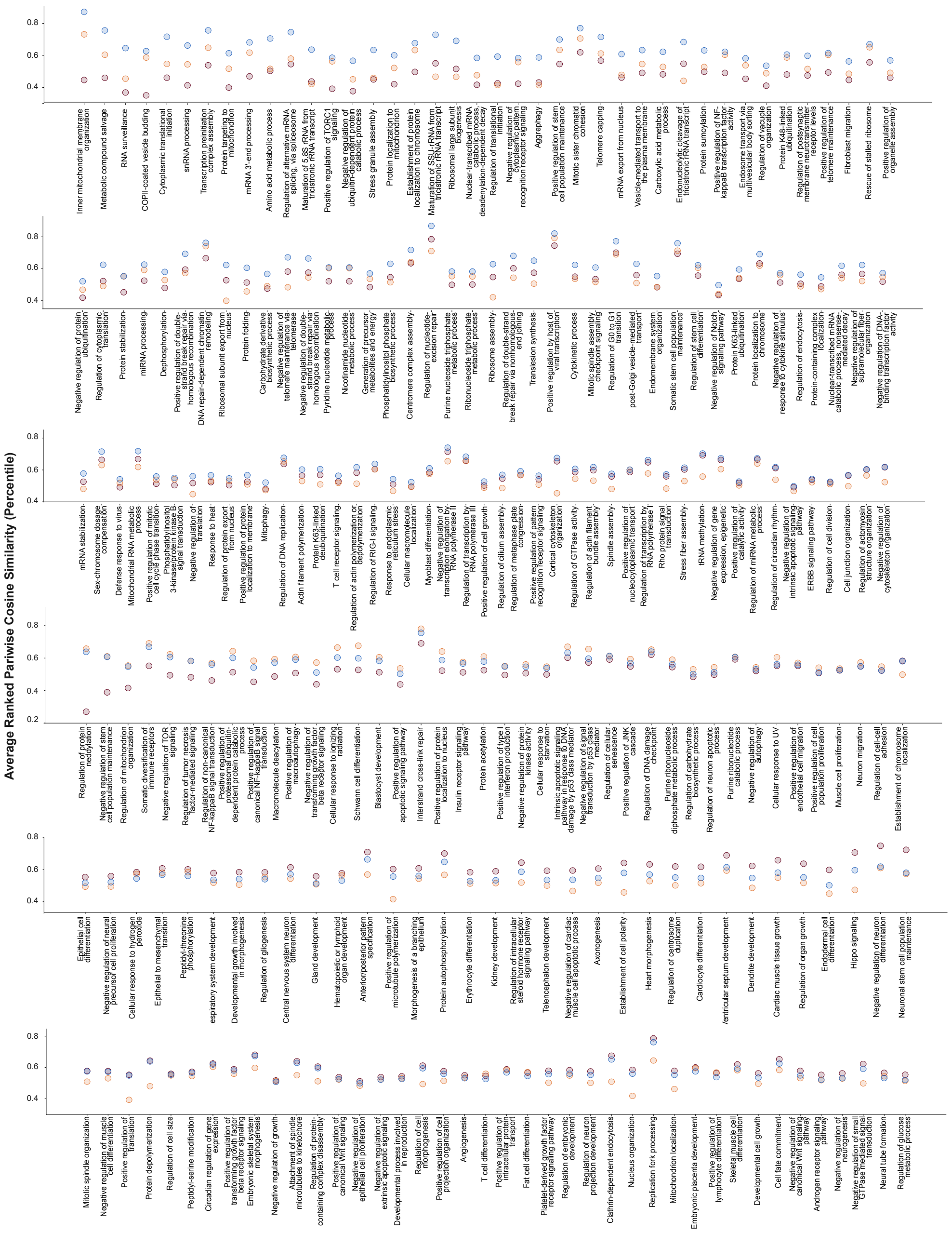

Fig. S5: Average embedding similarity (percentile of ranked similarity) of gene members across biological processes. Multimodal space values are shown in blue, sequence space values are shown in red, and image space values are shown in orange. The biological processes here are the set of those enriched in our gene set (described in the main section), excluding those shown in Figure 6. They are ordered by the difference between the multimodal value and the sequence value.

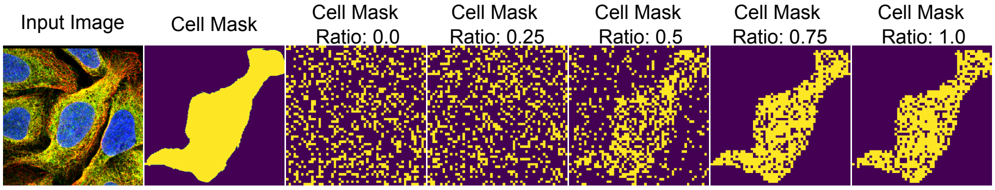

Fig. S6: Masking strategy used for removing the tokens in the MAEs using the cell masks. When the cell masking ratio is set to zero, the strategy follows random masking. As the ratio increases, more patches within the cell mask are randomly removed. The overall masking ratio was set to 0.25 for this image.

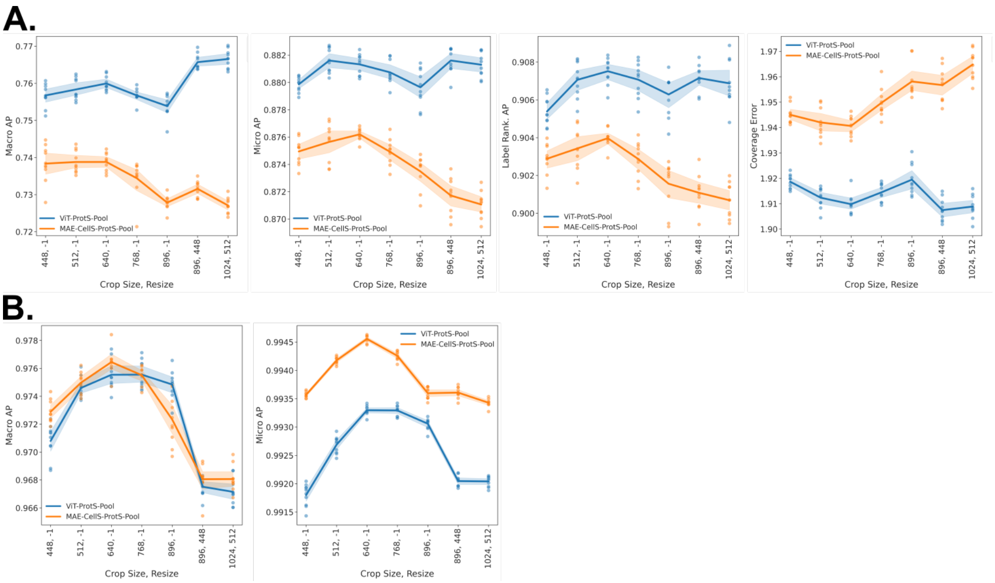

Fig. S7: The results of the classifiers trained on the features extracted by the models at different crop sizes and resolutions for A.) localization classification and B.) cell-line prediction tasks.

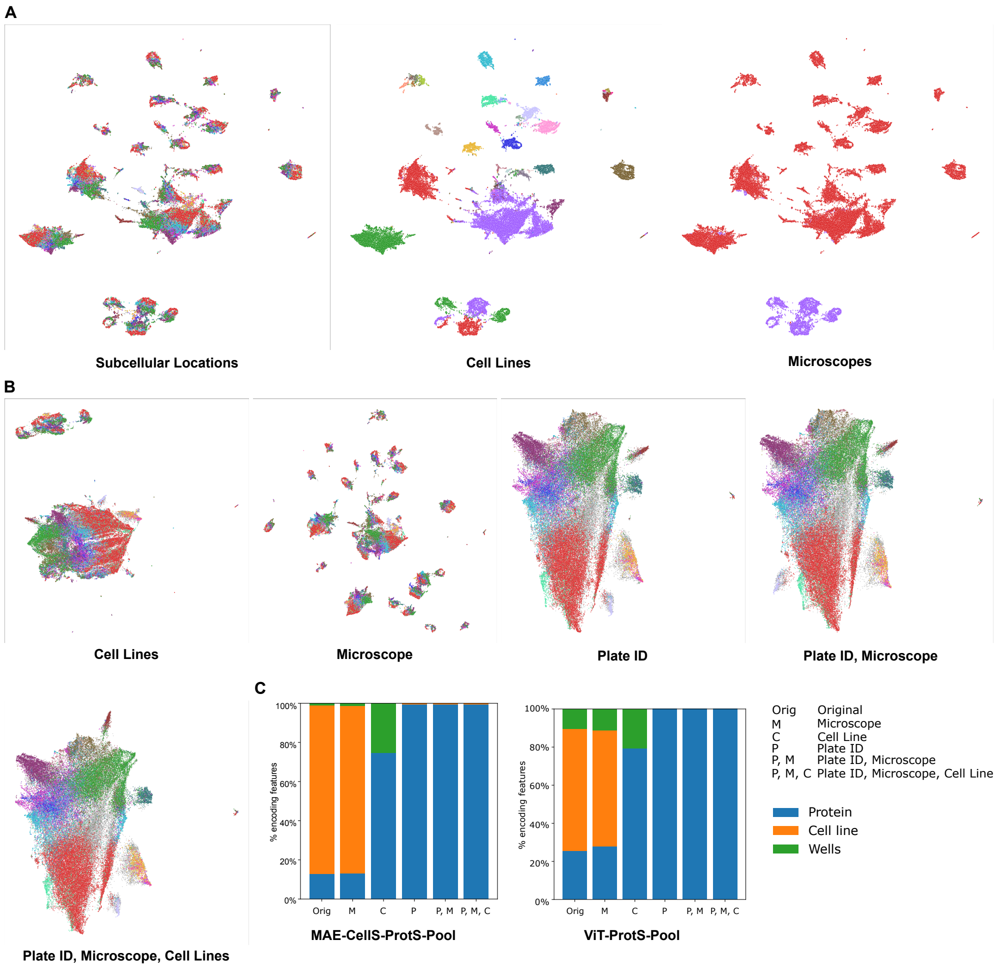

Fig. S8: Visualization of the feature space discovered by the SubCell models. A) UMAPs of the single-cell features extracted by the MAE-CellS-ProtS-Pool model aggregated by the FOVs colored by subcellular localizations (leftmost), cell lines (middle), and microscope (right). B) The FOV UMAPs showing the resulting features after integrating with Harmony over different factors of variations, namely, cell lines, microscopes, plate IDs, plate IDs and microscopes, and finally with plate IDs, microscopes, and cell lines, with colors representing the subcellular localization categories. C) Stacked-bar plot displaying the fraction of features that are strongly associated with three factors of variation annotated in the HPA dataset.

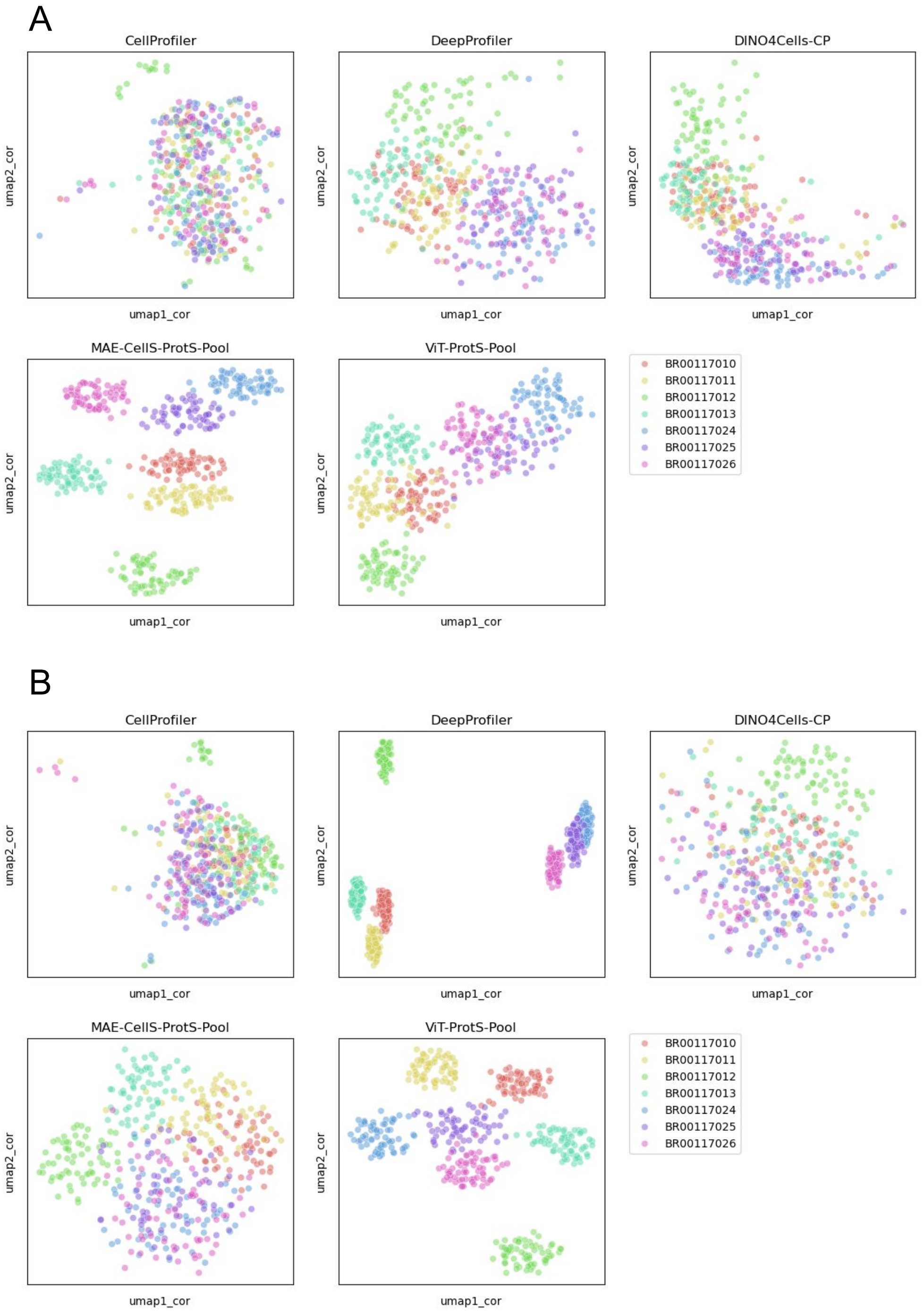

Fig. S9: UMAP visualization of the post-processed well-level profiles for each model that resulted in (A) the best compound mAP and (B) the best MoA mAP colored by plate. Only negative control wells are displayed.

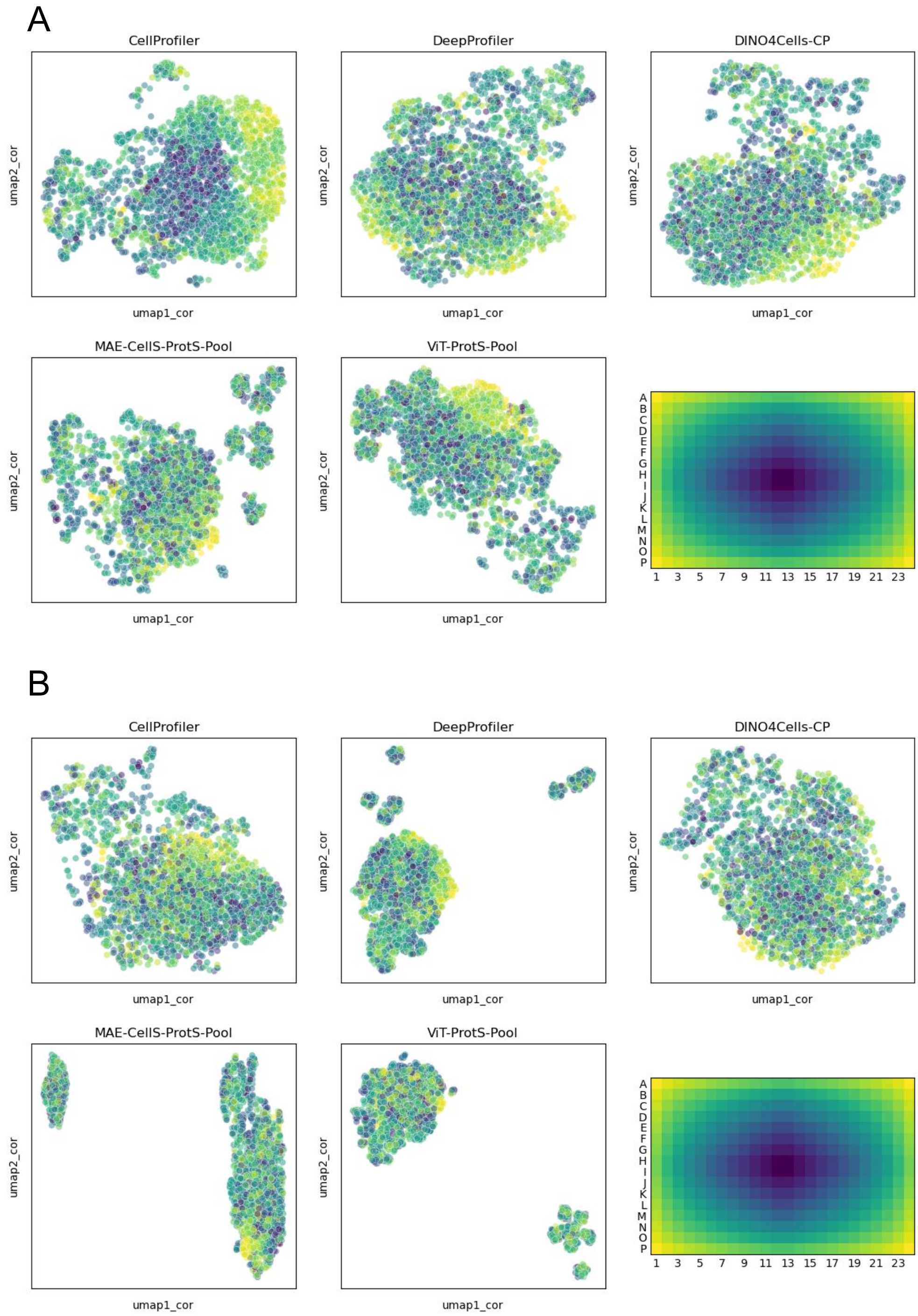

Fig. S10: UMAP visualization of the post-processed well-level profiles for each model that resulted in (A) the best compound mAP and (B) the best MoA mAP colored by well position. Color diagram of 384-well plate shown in the bottom right panel of both A and B.

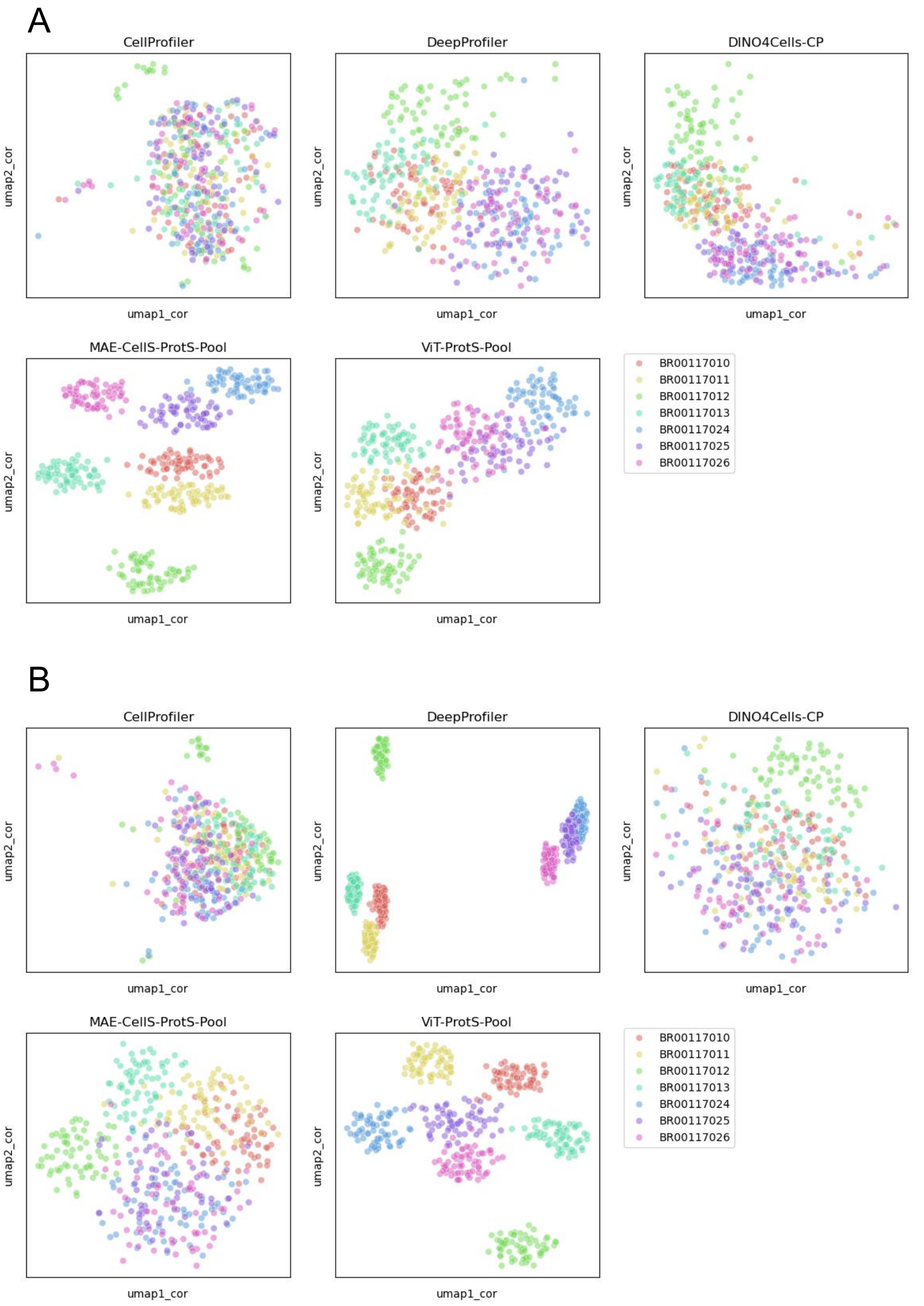

Fig. S11: UMAP visualization of the post-processed well-level profiles for each model that resulted in (A) the best compound mAP and (B) the best MoA mAP colored by well position. Color diagram of 384-well plate shown in the bottom right panel of both A and B.
